# Human Cytomegalovirus Vaccine Based on the Envelope gH/gL Pentamer Complex

**DOI:** 10.1371/journal.ppat.1004524

**Published:** 2014-11-20

**Authors:** Felix Wussow, Flavia Chiuppesi, Joy Martinez, John Campo, Erica Johnson, Christin Flechsig, Maegan Newell, Elaine Tran, Jose Ortiz, Corinna La Rosa, Andreas Herrmann, Jeff Longmate, Rana Chakraborty, Peter A. Barry, Don J. Diamond

**Affiliations:** 1 Department of Translational Vaccine Research, Beckman Research Institute of the City of Hope, Duarte, California, United States of America; 2 Department of Pediatrics, Emory University School of Medicine, Atlanta, Georgia, United States of America; 3 Department of Cancer Immunotherapeutics and Tumor Immunology, Beckman Research Institute of the City of Hope, Duarte, California, United States of America; 4 Department of Information Sciences, City of Hope, Duarte, California, United States of America; 5 Department of Pathology and Laboratory Medicine, University of California, Davis, Davis, California, United States of America; University of North Carolina at Chapel Hill, United States of America

## Abstract

Human Cytomegalovirus (HCMV) utilizes two different pathways for host cell entry. HCMV entry into fibroblasts requires glycoproteins gB and gH/gL, whereas HCMV entry into epithelial and endothelial cells (EC) requires an additional complex composed of gH, gL, UL128, UL130, and UL131A, referred to as the gH/gL-pentamer complex (gH/gL-PC). While there are no established correlates of protection against HCMV, antibodies are thought to be important in controlling infection. Neutralizing antibodies (NAb) that prevent gH/gL-PC mediated entry into EC are candidates to be assessed for *in vivo* protective function. However, these potent NAb are predominantly directed against conformational epitopes derived from the assembled gH/gL-PC. To address these concerns, we constructed Modified Vaccinia Ankara (MVA) viruses co-expressing all five gH/gL-PC subunits (MVA-gH/gL-PC), subsets of gH/gL-PC subunits (gH/gL or UL128/UL130/UL131A), or the gB subunit from HCMV strain TB40/E. We provide evidence for cell surface expression and assembly of complexes expressing full-length gH or gB, or their secretion when the corresponding transmembrane domains are deleted. Mice or rhesus macaques (RM) were vaccinated three times with MVA recombinants and serum NAb titers that prevented 50% infection of human EC or fibroblasts by HCMV TB40/E were determined. NAb responses induced by MVA-gH/gL-PC blocked HCMV infection of EC with potencies that were two orders of magnitude greater than those induced by MVA expressing gH/gL, UL128-UL131A, or gB. In addition, MVA-gH/gL-PC induced NAb responses that were durable and efficacious to prevent HCMV infection of Hofbauer macrophages, a fetal-derived cell localized within the placenta. NAb were also detectable in saliva of vaccinated RM and reached serum peak levels comparable to NAb titers found in HCMV hyperimmune globulins. This vaccine based on a translational poxvirus platform co-delivers all five HCMV gH/gL-PC subunits to achieve robust humoral responses that neutralize HCMV infection of EC, placental macrophages and fibroblasts, properties of potential value in a prophylactic vaccine.

## Author Summary

Human cytomegalovirus (HCMV) fetal infection during pregnancy and infection of immunocompromised patients are both clinical problems considered extremely important by the Institute of Medicine. Limited efficacy against primary HCMV infection was found using a subunit vaccine based on glycoprotein B, an important neutralizing antibody determinant blocking HCMV entry into fibroblasts. The HCMV field has been transformed by the discovery that a five-member (pentamer) protein complex is a required factor for epithelial and endothelial cell entry and indispensable for transmission as shown in non-human primates. Targeting HCMV with antibodies specific to the pentamer may interrupt horizontal and vertical transmission. We describe an innovative vaccine strategy to induce serum neutralizing antibodies of impressive magnitude against HCMV in two animal models. Using an attenuated poxvirus vector system, we demonstrate that co-expression of all five pentamer components is significantly more potent to induce serum neutralizing antibodies than subunit subsets of the complex or glycoprotein B, reaching peak levels comparable to HCMV hyperimmune globulin. A vaccine that elicits systemic and mucosal antibody responses that prevents infection of multiple cell types crucial to natural history of HCMV infection could play a role in preventing congenital HCMV infection and control of infection in immunocompromised patients.

## Introduction

HCMV infection causes morbidity and mortality in vulnerable hosts following horizontal or vertical transmission [1]. In immunosuppressed subjects, multi-system life-threatening disease can occur with primary infection, reinfection by a different HCMV strain, or after viral reactivation. HCMV is the most common congenital infection worldwide (0.2–2.0% of all pregnancies) often resulting in long-term consequences to the developing fetus including mortality [2], [3]. Intrauterine HCMV has a marked tropism for the developing central nervous system, and a consequence of congenital infection can be irrevocable neurological sequelae in newborns. Despite intensive research that spans four decades, there is no licensed vaccine to prevent HCMV infection [4], [5]. In 1999, the Institute of Medicine ranked HCMV infection in the highest category for vaccine preventable diseases because potential societal benefits from reduction in associated morbidity and mortality would far outweigh development costs [6]. Development of an HCMV vaccine that efficiently confers protection against primary HCMV infection or reinfection of a woman of child-bearing years by limiting horizontal transmission is a candidate solution to significantly reduce the devastating consequences of intrauterine HCMV infection [7].

A number of challenges have inhibited progress in HCMV vaccine development [8]. HCMV vaccine strategies have been guided, in large part, by historical precedents of currently licensed vaccines demonstrating that induction of pathogen-specific B cell immunity protects against infection and/or disease [9]–[11]. The disappointing Phase 2 trial showing that HCMV hyperimmune globulins are ineffective in the treatment of congenital HCMV infection has renewed interest in eliciting NAb by an HCMV vaccine [12]–[14]. Studies using fibroblasts as a cell substrate for infection have demonstrated that the envelope glycoprotein B (gB) is essential for HCMV entry [15]–[17]. gB elicits the majority of antibodies in immune individuals that neutralize fibroblast infection by blocking gB-mediated fusion between the virion and cell membrane [18]–[23]. Importantly, pregnant women who develop high avidity anti-gB NAb during primary infection are less likely to give birth to an infant with congenital infection compared to women who are initially CMV seronegative [24]–[26]. These observations formed the rationale to focus HCMV subunit vaccine design using gB [7], [27], [28]. A Phase II clinical trial based on recombinant gB admixed in the adjuvant MF59 established a protection level of 50% against primary HCMV infection among women who had given birth in the preceding year [29]. gB adjuvanted in MF59 is the sole example of a vaccine that has shown significant efficacy to protect healthy women against primary HCMV infection and seronegative solid organ transplant recipients from infection as a result of receiving a seropositive donor organ [30]. These clinical results provide evidence that a vaccine which targets major neutralizing epitopes may have a role in protection against primary HCMV infection. Consequently there is a strong interest to optimize vaccine strategies by identifying dominant viral targets of the host immune response that will improve protective efficacy [8].

While prior work highlights the importance of gB-specific immune responses in limiting primary infection, recent discoveries on viral entry conclude that NAb responses that prevent HCMV infection of various non-fibroblast cell types, including endothelial, epithelial cells (EC) and monocytes, are qualitatively different from those blocking fibroblast infection [31]–[33]. A current model proposes that HCMV entry into fibroblasts occurs by fusion at the plasma membrane and depends on envelope glycoprotein complexes composed of gB, gH/gL and/or gH/gL/gO [34]–[37]. Alternatively, HCMV entry into EC is mediated by endocytosis and requires an additional glycoprotein complex consisting of UL128, UL130, UL131A, and gH/gL, termed the gH/gL pentamer complex (gH/gL-PC)[33], [37]–[39]. Several studies provide evidence that gH/gL-PC is the dominant target of NAb in sera from naturally HCMV positive individuals that prevents HCMV infection of EC [31], [40]–[44]. Significantly, the majority of NAb in HCMV hyperimmune globulins (CMV-IVIg) are directed against epitopes of gH/gL-PC [44]. In contrast to NAb targeting epitopes of gB, gH/gL, or gM/gN that neutralize HCMV infection of fibroblasts or EC with moderate potency, NAb that recognize conformational epitopes of gH/gL-PC neutralize HCMV infection of EC with unusually high potency [41]. In addition, there is correlative evidence obtained by *in vitro* measurements for NAb responses to gH/gL-PC which have implications for the *in vivo* control of viral dissemination [45]. These data are consistent with previous studies showing that gH/gL-PC is an important target of the host response to HCMV infection, and induction of gH/gL-PC-specific NAb should be evaluated as a potential component of an HCMV vaccine trial.

We demonstrated that vaccination of rhesus macaques (RM) with a bacterial artificial chromosome (BAC)-derived MVA vector co-expressing all five rhesus cytomegalovirus (RhCMV) orthologs of HCMV gH/gL-PC elicits potent RhCMV-specific NAb responses [46]. Based on this observation, we constructed a single MVA that co-expressed all five HCMV gH/gL-PC subunits derived from the clinical-like isolate, TB40/E which has both EC and macrophage tropism [47]. This vector induced EC-specific NAb titers in mice and RM that were orders of magnitude higher than those induced by MVA expressing only gH/gL-PC subunit subsets (gH/gL or UL128/UL130/UL131A), or solely gB. In addition, MVA-gH/gL-PC elicited NAb preventing HCMV infection of fibroblasts with titers comparable to those induced by MVA expressing gH/gL or gB. NAb generated in RM by vaccination with MVA-gH/gL-PC also inhibited infection of placental macrophages called Hofbauer cells (HC) [48]. The avidity of CMV-specific antibodies durably rose after MVA-gH/gL-PC vaccination, indicating that the quality of responses is consistent with protective immunity. These results describe a novel vaccine strategy that induces high level NAb titers that interfere with two prominent HCMV entry routes utilizing a single vaccine vector, a possible requirement to prevent clinical HCMV infection.

## Results

### Generation of MVA vectors expressing gH/gL-PC subunit proteins or gB

**Figure 1:**
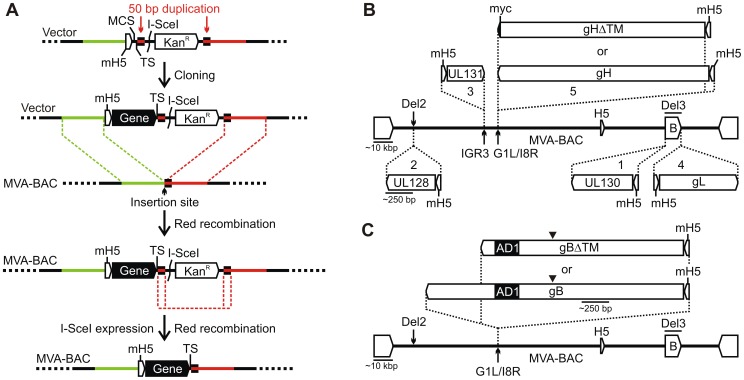
Insertion of gH/gL-PC subunits or gB into MVA-BAC. **A)** Gene insertion using transfer vector. The transfer vector contains in the indicated order a mH5 promoter, a multiple cloning site (*MCS*), and a transcription termination signal (TS). It further comprises a *I-SceI* recognition site and a kanamycin resistance marker (Kan^R^) both flanked by a 50 bp MVA sequence duplication (small black boxes). The entire construct is flanked by two ∼700 bp sequences of the MVA insertion site (large green and red bars). Following cloning, the gene is inserted into the MVA-BAC via a first Red recombination utilizing the large 700 bp sequence flanks. The selection marker is then removed after introduction of a double-strand break at the *I-SceI* site and a second Red recombination between the 50 bp sequence duplication. **B)** Gene insertion sites. HCMV genes were inserted with the indicated order (1-5) and 5′-3′ orientation into the MVA insertion sites Del2 (UL128), IGR3 (UL131), G1L/I8R (gH or gHΔTM, or at the ends of the BAC vector (**B**) within the Del3 insertion site (UL130 and gL). **C)** gB or gBΔTM were separately inserted into the G1L/I8R insertion site. Thin horizontal lines represent approximate lengths of either HCMV genes or portions of the MVA genome. Filled arrowheads in C indicate the cleavage site of the precursor gB protein encoded by the gB or gBΔTM gene cassette. AD1 designates antigenic domain 1, which is recognized by the anti-gB mAb 7-17[112].

We used as a model our recently described BAC-derived MVA vaccine vector for RhCMV gH/gL-PC, to rapidly and efficiently construct a single MVA vector expressing all five HCMV gH/gL-PC subunits (see Materials and Methods and Figure 1A for details) [46]. gH/gL-PC subunits were derived from HCMV TB40/E, a well-characterized clinical-like HCMV strain with an intact complement of gH/gL-PC genes [49]. Two versions of gH/gL-PC vectors were constructed expressing either a full-length version of gH (MVA-gH/gL-PC) or a variant of gH in which the transmembrane (TM) and cytoplasmic domains were deleted (MVA-gH/gL-PCΔ, Figure 1B). To provide a comparative basis for evaluating the NAb responses elicited by immunization with MVA-gH/gL-PC and MVA-gH/gL-PCΔ, MVA vectors expressing full-length gH/gL (MVA-gH/gL), UL128/UL130/UL131A (MVA-UL128-131), full-length gB (MVA-gB), and TM-deleted gB (MVA-gBΔ) were also constructed (Figure 1B and 1C). gO is known to associate with gH/gL and to contribute to the endoplasmic reticulum transport of these proteins, anchoring either gH/gL or gH/gL/gO complexes within the virion envelope [50], [51]. We did not investigate co-expression of gH/gL and gO because gO does not promote gH/gL cell surface expression, and is therefore unlikely to aid in more effectively displaying gH/gL to the immune system [36]. We also did not test a combination of the gH/gL-PC and gO, since gO would likely compete with UL128, UL130 and UL131A subunits to associate with gH/gL and, thus, prevent or decrease the formation of gH/gL-PC and the induction of potent EC specific NAb responses[36], [50], [51]. Full-length gH or gB ORFs with an intact TM domain as well as their TM-deleted versions were inserted between the essential ORFs G1L and I8R of the MVA genome, an insertion site that is known to provide stable propagation of large and/or unstable sequences (Figure 1B and 1C)[52]. Vectors to express full-length or TM-deleted gH and gB were constructed to test which complex, a membrane-tethered or a soluble one would elicit the strongest NAb responses. Deletion of the TM domain of gB has been shown to improve the protein expression and the generation of NAb when compared to its full-length counterpart [53], [54]. MVA recombinants were reconstituted in baby hamster kidney (BHK) cells [46], and expanded to generate virus stocks for *in vitro* and *in vivo* characterization.

### Expression of gH/gL-PC or gB subunits from MVA

**Figure 2:**
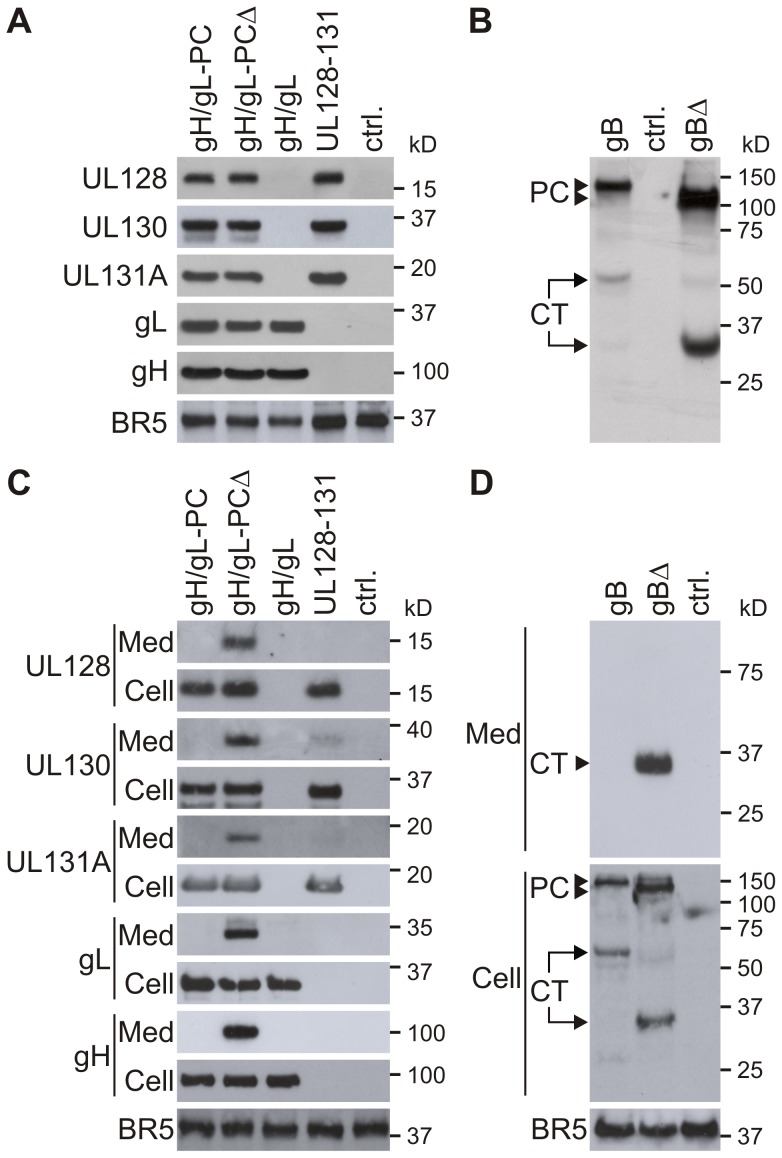
Expression of gH/gL-PC subunits and gB from MVA. Whole cell lysates of BHK cells or whole cell lysates and concentrated serum-free medium of CEF cells infected with the indicated MVA recombinants were analyzed by Immunoblot. gH/gL-PC subunits were detected with anti-HCMV gH mAb 11-1-1 and rabbit polyclonal antisera to UL128, UL130, UL131A, and gL. gB proteins were detected with anti-gB HCMV mAb 7-17, recognizing the C-terminal fragment of gB (Figure 1C). **A and B)** Immunoblot detection of gH/gL-PC subunits (A) or gB proteins (B) in MVA-infected BHK cells. **C and D)** Immunoblot detection of gH/gL-PC subunits (C) or gB proteins (D) in cell lysates (Cell) and concentrated medium (Med) of CEF cells infected with the MVA recombinants. MVA expressing the fluorescent marker Venus was a control (ctrl.) in A-D. Vaccinia virus BR5 was detected in the samples as loading control. PC  =  precursor protein; CT  =  C-terminal cleavage product of gB.

HCMV proteins that were expressed from all gH/gL-PC-related vectors displayed sizes in agreement with published values (Figure 2A)[33], [39], [55], [56]. There was no discernible size difference between full-length gH (MVA-gH/gL-PC and MVA-gH/gL) and truncated gH (MVA-gH/gL-PCΔ), since the size difference of the TM-deleted gH (ΔgH) and its full-length counterpart was only fourteen amino acids (AA) taking into account deletion of the TM and addition of the myc-tag (Figure 1B). Comparable levels of expression for each of the gH/gL-PC components were found from either the MVA-gH/gL-PC or MVA-gH/gL-PCΔ vectors (Figure 2A). Expression levels of UL128, UL130, and UL131A appeared to be slightly greater in MVA-UL128-131 than in either MVA-gH/gL-PC or MVA-gH/gL-PCΔ. Of note, the MVA-expressed BR5 antigen [57] used as a loading control had slightly higher expression in both MVA-UL128-131 and MVA expressing the fluorescent marker Venus [58] than any of the gH expressing MVA vectors (Figure 2A). These small differences in expression levels have not been explored further, and may reflect properties of MVA and/or the protein subunits. Full-length gB harvested from whole cells shows a 130 kDa precursor (PC) band, and a 55 kDa band corresponding to the processed C-terminal (CT) fragment (Figure 2B). The 110 kDa precursor form and a truncated C-terminal (CT) cleavage product (35 kDa) were detectable in MVA-gBΔ infected cells (Figure 2B). In summary, these data show that MVA-BAC technology allows rapid generation of MVA expressing all five gH/gL-PC subunits with either gH or gHΔ, subunit subsets (UL128-UL131A or gH/gL), gB or gBΔ proteins with sufficient stability for animal vaccination.

### Secretion of gH/gL-PC subunits or gB depends on transmembrane deletion

We first investigated if deletion of the TM domain of gH led to secretion of the gH/gL-PC subunits by performing immunoblot analysis of concentrated serum-free medium from chicken embryo fibroblasts (CEF) which are permissive for MVA infection. As observed in infected BHK cells (Figure 2A), comparable levels of the individual gH/gL-PC subunits were detectable in whole cell lysates of CEF cells infected with MVA expressing subsets or all 5 gH/gL-PC subunits (Figure 2C;Cell). In contrast, efficient secretion of all 5 gH/gL-PC subunits was only detectable in the medium of CEF cells infected with MVA-gH/gL-PCΔ (without gH TM); whereas none of the subunits were detected in the medium of MVA-gH/gL-PC (with gH TM) infected CEFs (Figure 2C; Med). In addition, gH or gL was not detectable in the medium of MVA-gH/gL (with gH TM) infected CEFs. In the case of MVA-UL128-131, only small amounts of UL130 and UL131A were observed in the medium of MVA-UL128-131 infected cells, though UL128 was undetectable (Figure 2C). These data indicate that deletion of the gH TM promotes efficient secretion of all 5 gH/gL-PC subunits, whereas maintenance of the gH TM retains the gH/gL-PC subunits or gH/gL in the cell or on the cell surface when co-expressed from MVA. Therefore, we believe that the data implicates the gH TM as a pivotal structure for the co-localization of all five gH/gL-PC subunits on the cell surface, and in the absence of the gH TM, the complex is secreted into the medium from the MVA-infected cell (Figure 2C). Presumably, the scaffold properties of the gH protein allow assembly of the full five member complex anchored to the cell surface. We also investigated the impact of TM deletion on secretion of the gB protein. Protein species corresponding to the precursor and the C-terminal fragments of gB and gBΔTM in lysates of MVA-gB or MVA-gBΔ infected CEFs were similar to those found in BHK cells (Figure 2B and D; Cell). However, only the cleavage product of the gBΔTM protein was detectable in the medium from gBΔ-MVA-infected CEF (Figure 2D, Med). We hypothesize that the gB TM tethers the full-length or precursor forms of gB onto the cell surface which limits the secretion of these forms of gB from MVA-gB infected cells.

### Complex formation of gH/gL-PC subunits expressed from MVA

**Figure 3:**
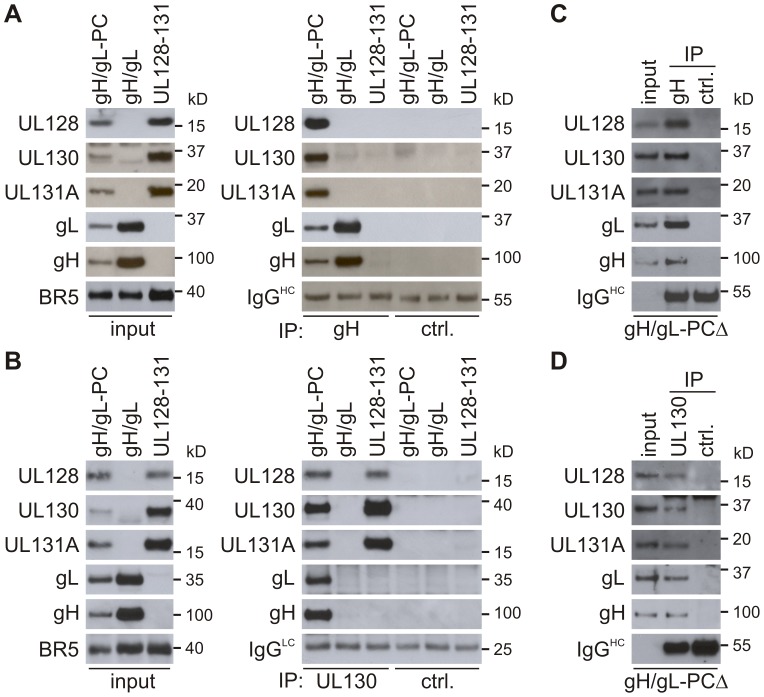
Complex formation of gH/gL-PC subunits co-expressed from MVA. Cell lysates of BHK cells (prepared under non-denaturing conditions) or concentrated serum-free medium of CEF infected with MVA recombinants were subjected to co-IP with anti-HCMV gH mAb 11-1-1 or UL130 polyclonal antiserum. Immunoprecipitated gH/gL-PC subunits were detected with anti-gH mAb 11-1-1 and UL128, UL130, UL131A, and gL rabbit polyclonal antisera. **A and B)** Immunoblot detection of gH/gL-PC subunits after co-IP from BHK cells infected with MVA-gH/gL-PC, MVA-gH/gL, or MVA-UL128-131. **C and D)** Immunoblot detection of gH/gL-PC subunits following co-IP from concentrated medium of MVA-gH/gL-PCΔ-infected CEF cells. Irrelevant mouse IgG Ab and rabbit UL130 pre-immune serum were used for IP controls (ctrl.). IgG^HC^ or IgG^LC^  =  Immunoglobulin G heavy or light chains. Input in A-D  =  detection of gH/gL-PC subunits in the samples subjected to co-IP.

We examined if multi-protein complexes were formed by expression of gH/gL-PC from MVA-gH/gL-PC, gH/gL expressed from MVA-gH/gL or UL128-UL130-UL131A expressed from MVA-UL128-131, and characterized their subunit composition using co-immunoprecipitation (co-IP) with two different antibody (Ab) preparations. Using the monoclonal antibody (mAb) 11-1-1 to HCMV gH [59] to isolate protein complexes containing gH, all five gH/gL-PC subunits were immunoprecipitated from BHK cells infected with MVA-gH/gL-PC, and gH/gL proteins were immunoprecipitated from cells infected with MVA-gH/gL (Figure 3A). As expected, the UL128-UL131A proteins expressed from MVA-UL128-131 were not immunoprecipitated by the anti-gH mAb. Similar to the gH antibody results, polyclonal antibodies to UL130 immunoprecipitated all five gH/gL-PC subunits from BHK cells infected with MVA-gH/gL-PC (Figures 3A and B). As expected, gH/gL protein complexes expressed from MVA-gH/gL were not immunoprecipitated by UL130 polyclonal antibodies, though UL128, UL130 and UL131A proteins expressed from MVA-UL128-131 were immunoprecipitated by UL130 antisera (Figure 3). In comparison to the immunoprecipitated proteins from MVA-gH/gL-PC, higher amounts of gH/gL were confirmed following IP of gH from MVA-gH/gL infected cells, and of UL128, UL130, and UL131A after IP of UL130 from cells infected with MVA-UL128-131. This difference in immunoprecipitated gH/gL or UL128-131A proteins can be explained by differing protein amounts extracted from MVA-gH/gL-PC infected cells in comparison to proteins extracted from cells infected with MVA-gH/gL or MVA-UL128-131, respectively (input; Figure 3A and 3B). HCMV proteins were not immunoprecipitated by an IgG control Ab (Figure 3A) or rabbit polyclonal pre-immune serum (Figure 3B), confirming the specific co-IP of individual gH/gL-PC subunits by the gH mAb or UL130 antiserum. The major conclusions from these observations were that (1) four other gH/gL-PC subunits associate either directly or indirectly with gH or UL130 and are pulled down with anti-gH or anti-UL130 antibodies when co-expressed from MVA-gH/gL-PC, (2) gL interacts with gH when co-expressed from MVA-gH/gL, and (3) UL128-UL130-UL131A interact with each other when expressed by MVA-UL128-131. Together, these data indicate that all three MVA constructs can express HCMV proteins capable of forming complexes, at least when cell-associated.

### Secretion of gH/gL-PCΔ complexes from MVA-gH/gL-PCΔ infected BHK cells

An important result would be to demonstrate that the subunits that compose gH/gL-PCΔ physically associate with each other to form a secreted complex. To determine if the complex that was released into the medium of cells infected with MVA-gH/gL-PCΔ contained all five gH/gL-PC subunits, we performed co-IP using concentrated medium harvested from MVA-gH/gL-PCΔ infected CEF. IP was carried out either using mAb 11.1.1 to gH (Figure 3C) or rabbit polyclonal antiserum to UL130 (Figure 3D), with a similar result that all 5 subunits were shown to associate by co-IP. While the gH mAb was more efficient in detecting and precipitating the gH/gL-PC than the polyclonal antiserum to UL130, the qualitative result was the same, which shows evidence that the secreted form of the gH/gL-PCΔ is maintained as an intact complex in the cell culture medium.

### TM-dependent cell surface expression of gH/gL-PC

**Figure 4:**
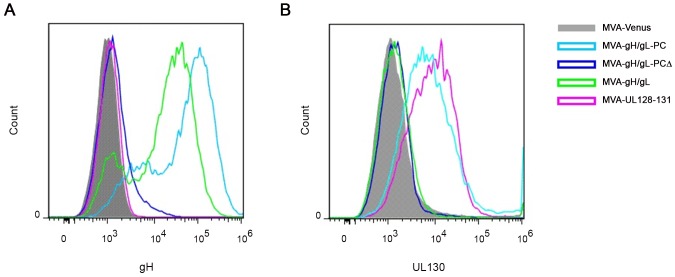
Cell surface detection of gH and UL130 expressed from MVA recombinants. BHK cells infected with MVA recombinants were analyzed by FC for cell surface staining of gH or UL130. Staining was performed with mouse anti-HCMV gH mAb 14-4b or UL130 rabbit antiserum as primary antibodies and fluorophore-coupled secondary Ab anti-mouse or anti-rabbit Alexa Fluor 647. **A)** FC analysis of cell surface staining of gH on BHK cells infected with the indicated gH/gL-PC-related vectors. **B)** FC analysis of cell surface staining of UL130 on BHK cells infected with the indicated gH/gL-PC-related vectors. Cells infected with MVA-Venus were analyzed as a control.

Next, we investigated whether the efficient transport of gH complexes (gH/gL-PC, gH/gL-PCΔ, or gH/gL) to the cell surface depends on the TM of the gH subunit (Figure 4A). BHK cells infected with MVA recombinants were analyzed by flow cytometry (FC) using the anti-gH Ab 14-4b to evaluate cell surface expression of the gH subunit with or without its TM domain. Cells infected with MVA-gH/gL-PC showed higher expression of cell-surface gH than other infected samples and the control cells (Figure 4A). The minimal detection of cell-surface gH on MVA-gH/gL-PCΔ-infected cells (Figure 4A), may reflect transit of soluble complexes through the plasma membrane, rather than actual cell-surface expression. As expected, cell surface expression of gH was detected on MVA-gH/gL-infected cells, although at a lower density than on MVA-gH/gL-PC infected cells, consistent with previous observations that the transport of gH to the cell-surface is more efficient when all 5 gH/gL-PC subunits are co-expressed in comparison to only gH and gL [39]. We confirmed the FC results that we obtained with the anti-gH mAb by using the same rabbit polyclonal Ab preparation specific for UL130 that was used in the co-IP experiments in Figure 3. Cell surface UL130 antigen was detected on MVA-gH/gL-PC infected cells, although not on cells infected either with MVA-gH/gL-PCΔ or MVA-gH/gL (Figure 4B). UL130 was also detectable on cells infected with MVA-UL128-131 with comparable density to that observed for MVA-gH/gL-PC (Figure 4B). GFP expression quantified by its fluorescence properties was used to demonstrate equal infection of cells by all MVA vectors examined in Figure 4A and B (data not shown). BAC derived MVA expressed GFP due to the vector construction [60]. Collectively, these results indicate that gH and UL130 are transported to the cell surface when all five gH/gL-PC subunits are co-expressed from MVA when gH contains a TM. Further proof is needed to establish if gH and UL130 are expressed on the cell surface in a complex with UL128, UL131A, and gL. Nonetheless, as shown by the secretion experiment in Figure 3C and D, complexes composed of all five gH/gL-PC subunits are secreted and not cell-surface associated when the gH TM is deleted. Further work is needed to explain how UL130 is transported to the cell surface in the absence of gH/gL.

### IF demonstrates cellular localization of gH/gL complexes is dependent on presence of TM

We utilized immunofluorescence (IF) to detect surface expression of gH on BHK cells infected with MVA-gH/gL-PC, MVA-gH/gL-PCΔ or MVA-gH/gL by staining with anti-gH HCMV mAb 14-4b. We permeabilized MVA-infected cells to facilitate intracellular Ab penetration. This enabled labeling of internal cytoplasmic proteins to compare with the results of Ab labeling of non-permeabilized cells. Consistent with the FC data (Figure 4A), cell surface staining of gH without permeabilization was only observed for constructs expressing TM containing glycoprotein complexes (MVA-gH/gL-PC and MVA-gH/gL Figure S1; non-permeabilized). In contrast, only a few cells infected with MVA-gH/gL-PCΔ showed staining for gH with less intensity when compared to MVA-gH/gL-PC infected cells, which may reflect TM-deleted gH protein transitioning through the plasma membrane or intracellular penetration of the gH Ab through leaky cell membranes (Figure S1). However, when cells infected with any of the three constructs were permeabilized, the signal was brighter and emanated prominently from the cytoplasm. The effect is most prominent in the case of MVA-gH/gL-PCΔ, but clearly visible with either MVA-gH/gL or MVA-gH/gL-PC infected cells. GFP expression was monitored to localize areas of viral foci. As shown in the control panel (Figure S1), most nuclei were intensely stained with an anti-Histone 3 Ab when cells were permeabilized, while only a few scattered nuclei were stained with less intensity in non-permeabilized cells. This indicates that only permeabilization allowed intracellular Ab entry, whereas non-permeabilized cells were resistant to Ab entry. We were unable to effectively use the UL130 polyclonal antiserum for IF studies to replicate the results we found with the anti-gH mAb; therefore we cannot conclude that UL130 is present on the cell-surface alone or as part of a protein complex using the IF approach (data not shown). Direct evidence that gH with a TM is associated with the other four gH/gL-PC subunits will require further study with Ab reagents that bind to cell-surface forms of these proteins.

### gH/gL-PC subunits co-expressed from MVA elicit murine NAb blocking HCMV infection of EC

**Figure 5:**
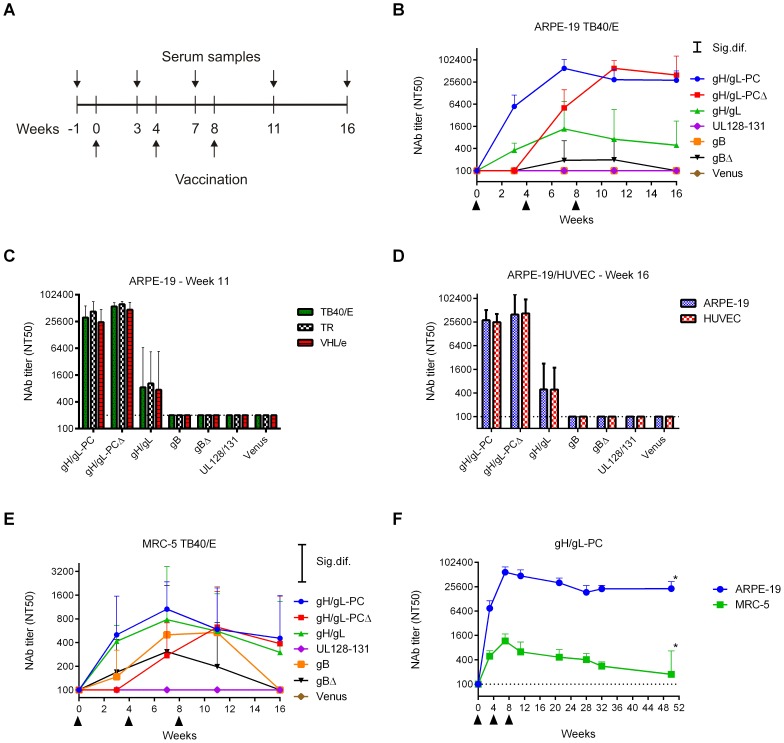
NAb induction by MVA recombinants in mice. Balb/C mice were vaccinated three times by intraperitoneal (i.p.) route with MVA recombinants. HCMV-specific serum NAb titer (NT50) was determined at different time points post-vaccination. **A)** Schematic of time line for MVA vaccinations and serum sample collection. **B-D**) Shown are NT50 titers (geometric mean titer (GMT); n = 4) of vaccine groups measured at different time points on ARPE-19 cells against TB40/E (**B**), at week 11 on ARPE-19 cells against different HCMV strains (TB40/E, TR, VHL/e) (**C**), or at week 16 on ARPE-19 cells and HUVECs against TB40/E (**D**). **E**) NT50 titers measured on MRC-5 fibroblasts against TB40/E in the same samples as in B. **F)** Serum NT50 titers in MVA-gH/gL-PC-vaccinated mice (GMT; n = 8) measured on ARPE-19 and MRC-5 cells against TB40/E over one year (n = 4 at week 50 (asterisks)). Significance bars (Sig. dif.) in B and E indicate points of significance at *p* = 0.05. Dotted lines in all panels indicate the detection limit of the assay. Upper bars represent 95% confidence intervals.

Vaccination of mice using MVA recombinants was performed to evaluate which combination of HCMV antigens induced NAb that prevented *in vitro* HCMV infection of ARPE-19 epithelial cells and human umbilical vein endothelial cells (HUVEC). Mice were vaccinated three times (0, 4, and 8 weeks) by the intraperitoneal (i. p.) route (Figure 5A) and the NAb titer that blocked 50% infection (NT50) of HCMV strain TB40/E was evaluated on human ARPE-19 cells [49]. Marked differences were noted in the ability of different vectors to induce NAb. Only MVA-gH/gL-PC and MVA-gH/gL-PCΔ stimulated high NAb titers that blocked TB40/E infection of ARPE-19 cells (peak titers >62,000; Figure 5B and Table S1). NAb titers in MVA-gH/gL-PC vaccinated mice were detectable after the initial vaccination, increased to maximum NT50 levels after the first boost, and only slightly declined following the second boost (Figure 5B and Table S1). However, the kinetics of reaching peak NAb titers in MVA-gH/gL-PCΔ vaccinated mice were quite different. NAb were undetectable following the initial vaccination, but increased to maximum NT50 levels after two boosts (peak titers >62000; Figure 5B and Table S1). Substantially lower titers were observed with MVA-gH/gL (peak titer = 2,170) and lower yet with MVA-gBΔ (peak titer = 250). No EC NAb were stimulated with MVA-UL128-131 or MVA-gB (Figure 5B). In a repeat experiment examining MVA-gH/gL-PC, high titer NAb were stably elevated over a period of at least fifty weeks after initial vaccination (Figure 5F). In experiments conducted with HUVECs using the identical methods, we found almost identical results as documented on ARPE-19 cells (Figure 5B-D, F) using week seven (data not shown) and week sixteen (Figure 5D) serum. Both gH/gL-PC expression constructs, either with or without gH TM, induce comparable peak NAb levels, but these titers are more rapidly induced with a membrane-anchored form than with a TM deleted form of the gH/gL-PC. The impact of the TM on the kinetics of the NAb response is noteworthy as it may reflect a reduced capacity by the immune system to generate a rapid humoral response to a secreted form versus the cell surface form of the gH/gL-PC. Ultimately, after three vaccinations, both forms of the complex support the development of equivalent levels of NAb.

### MVA- gH/gL-PC vaccinated mice generate immune sera that detect heterologous HCMV strains

Since UL128-UL131A are highly conserved proteins among different HCMV strains [61], NAb recognizing them should neutralize different HCMV strains with comparable potency. We performed neutralization assays on ARPE-19 cells using the TB40/E, TR [62] and VHL/e [63], [64] strains of HCMV for infection. The five proteins that compose the gH/gL-PC of TB40/E and TR have protein sequences which are >97% identical (Accession # EF999921 and KF021605). Sequence information for the clinical-like VHL/e strain is unavailable. There is sufficient sequence variability in the gH amino acid sequence to define two separate clusters of gH sequences designated as gH-group 1 and group 2 (Figure S2). The strains that we investigated in Figure 5C are each assigned to a separate gH group (Figure S2). Yet, serum antibodies generated against TB40/E proteins expressed from MVA neutralized group 1 (TR) and 2 (TB40/E) gH strains equivalently (Figure 5C). In fact, MVA-gH/gL-PC and MVA-gH/gL-PCΔ induced equivalent NAb titers against all three HCMV strains, reaching NT50 levels >30,000 (Figure 5C). Similarly, MVA-gH/gL elicited comparable NAb titers against all three strains, although all were markedly lower than those generated following immunization with either MVA-gH/gL-PC or MVA-gH/gL-PCΔ (Figure 5C). MVA-UL128-131, MVA-gB, or MVA-gBΔ did not stimulate detectable EC-specific NAb in this experiment. These data indicate that MVA-gH/gL-PC and MVA-gH/gL-PCΔ elicit high titer NAb, and far greater than MVA-gH/gL to prevent HCMV infection of EC with heterologous HCMV isolates with limited gH/gL-PC sequence diversity.

### Co-expressed gH/gL-PC subunits induce NAb that prevent HCMV infection of fibroblasts

We discovered that an MVA expressing all five RhCMV orthologs of HCMV gH/gL-PC induced robust NAb responses that prevented RhCMV infection of rhesus EC and fibroblasts [46]. That was the premise for investigating if the mice that were vaccinated with MVA expressing HCMV gH/gL-PC developed NAb that blocked HCMV TB40/E infection of MRC-5 fibroblasts. Measurable NAb titers were generated following vaccination of mice with all MVA recombinants, except MVA-UL128-131 (Figure 5E and Table S1). NAb induced by MVA-gH/gL-PC and MVA-gH/gL were comparable in titer and kinetics (peak titers >1000; Table S1). NAb titers induced by MVA-gH/gL-PC were stable over a period of twenty weeks following the second boost (Figure 5F). In contrast, NAb titers from MVA-gH/gL-PCΔ infected mice were undetectable after the initial vaccination, but increased to levels comparable to those induced by MVA-gH/gL-PC in response to two boosts (Figure 5E and Table S1). NAb titers of mice vaccinated with MVA-gB kept pace with MVA-gH/gL-PC vaccinated mice, but titers dropped to baseline at the conclusion of the experiment (sixteen weeks). Mice vaccinated with MVA-gBΔ developed NAb titers (peak titers = 510) that were lower than all other vaccine groups except MVA-UL128-131 which failed to induce any measurable NAb titer, identical to its behavior in the EC system (Figure 5B and Table S1). Collectively, these results emphasize the biologic importance of the MVA-gH/gL-PC vaccine and highlight immunologic differences in potency of sera from mice vaccinated with different MVA constructs to neutralize HCMV infection of fibroblasts and EC.

### MVA-gH/gL-PC induces high NAb titers in vaccinated RM: EC neutralization

**Figure 6:**
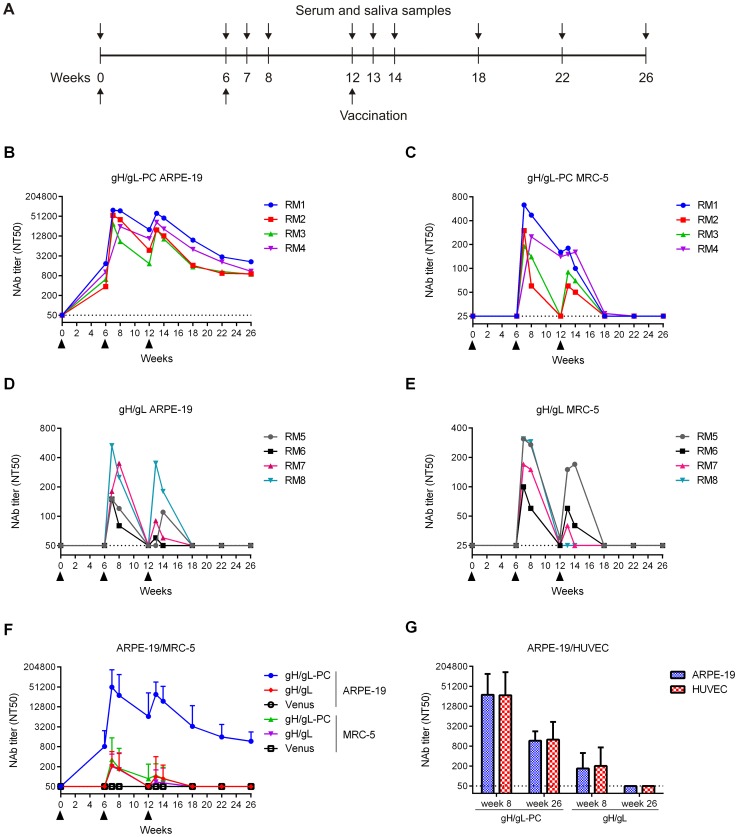
HCMV-specific serum NAb titers in vaccinated RM. Groups of four RM were vaccinated three times with MVA recombinants by intramuscular (i.m.) injection. HCMV-specific serum NT50 titers were determined at multiple time points on ARPE-19 cells, MRC-5 fibroblasts and HUVEC using HCMV TB40/E for infection. **A)** Schedule for vaccination and sample preparation. **B-E)** Serum NT50 titer of individual RM vaccinated with MVA-gH/gL-PC (B, C) or MVA-gH/gL (D, E) measured on ARPE-19 and MRC-5 cells. **F)** Comparison of serum NT50 titers determined in MVA-gH/gL-PC or MVA-gH/gL vaccine groups (GMT; n = 4) on ARPE-19 and MRC-5. Serum from RM vaccinated with MVA-Venus was analyzed as a control. **G)** Serum NT50 titer in vaccine groups (GMT, n = 4) measured on ARPE-19 cells and HUVECs at weeks 8 and 26 post-initial vaccination. Filled arrowheads indicate vaccinations. Dotted lines represent detection limits. Upper bars represent 95% confidence intervals.

We performed vaccinations studies in RM to investigate NAb induction by MVA-gH/gL-PC or MVA-gH/gL in an animal model that is evolutionarily closely related to humans. RhCMV-uninfected RM were immunized three times with MVA-gH/gL-PC, MVA-gH/gL or MVA-Venus (four RM per group) (Figure 6A). We chose MVA-gH/gL-PC instead of MVA-gH/gL-PCΔ because the full-length version of gH/gL-PC produced higher titer NAb with faster kinetics than with TM-deleted gH in our mouse experiments (Figure 5B and E). All MVA vaccines were well-tolerated with no evidence of local injection site or systemic reaction (data not shown). Both MVA-gH/gL and MVA-gH/gL-PC stimulated EC- and fibroblast-specific NAb responses in RM, and, as observed following vaccination in mice, notably higher EC titers were elicited with MVA-gH/gL-PC than with MVA-gH/gL (Figure 6B-F; Table S2). EC-specific NAb were detected in all four vaccinated RM at the time of the first booster immunization with MVA-gH/gL-PC (six weeks), and anamnestic responses were observed after the booster immunizations at weeks six and twelve (Figure 6B and F). Memory NAb responses were stimulated after two booster immunizations with MVA-gH/gL (Figure 6D and F), but peak titers (150–530; Table S2) were two orders of magnitude less than those generated with MVA-gH/gL-PC (29,960–78,340, Table S2). NAb titers declined for both MVA-gH/gL-PC and MVA-gH/gL, but one distinction for MVA-gH/gL-PC was that NAb titers declined to a plateau, and remained elevated fourteen weeks after the second booster at week twelve (Figures 6B and F, Table S2). In contrast, EC NAb titers declined to background over the same time frame in animals immunized with MVA-gH/gL (Figures 6D and F). Progressive declines in NAb titers down to the limit of detection are a common observation in RM vaccinated with DNA vaccines or viral vectors [43], [46], and the sustainability of EC-specific NAb after MVA-gH/gL-PC vaccination is a notable exception to this pattern. We also investigated if serum from RM that was active on ARPE-19 cells could neutralize HCMV infection of HUVECs (Figure 6G). Similar to the case of immune mouse serum (Figure 5D), NT50 titers on HUVECs were almost identical to what we measured on ARPE-19 cells, showing that neutralization extends to both epithelial and endothelial cell types with an almost identical pattern and sensitivity.

### MVA-gH/gL-PC induces high NAb titers in vaccinated RM: Fibroblast neutralization

Sera from monkeys vaccinated with MVA-gH/gL-PC or MVA-gH/gL were tested for NAb that prevented HCMV TB40/E infection of MRC-5 fibroblasts. As observed in mice, MVA-gH/gL-PC and MVA-gH/gL induced comparable fibroblast-specific NAb titers in vaccinated RM (peak titers 190–630 and 100–310, respectively) (Figures. 6C, E, and F; Table S2). Two injections were required to stimulate detectable NAb with both vectors. As with NAb that interfered with HCMV infection of EC, comparable NAb interfering with fibroblast infection progressively declined after the first and second booster. While the second booster stimulated NAb titers, peak titers after the second boost did not reach those observed after the first boost, and NAb titers were below or at the level of detection within six weeks of the second boost (eighteen weeks). These data confirm in RM that MVA-gH/gL-PC induces NAb that prevent HCMV infection of fibroblasts with levels comparable to those induced by MVA-gH/gL. Moreover, these data extend our mouse results in a primate host and demonstrate that MVA expressing all five gH/gL-PC subunits induce high titer HCMV-specific NAb. Detection of ARPE-19 specific NAb titers that exceed those measured on fibroblasts is consistent with observations made with HCMV-positive human sera [44], [65]. We note that NAb titers measured on fibroblasts are significantly higher using MVA constructs expressing gH/gL or gH/gL-PC than control sera. Nevertheless, they are far lower than those measured for ARPE-19 and HUVEC cells using identical sera.

### MVA-gH/gL-PC induced higher NAb titer than HCMV positive serum preparations

**Figure 7:**
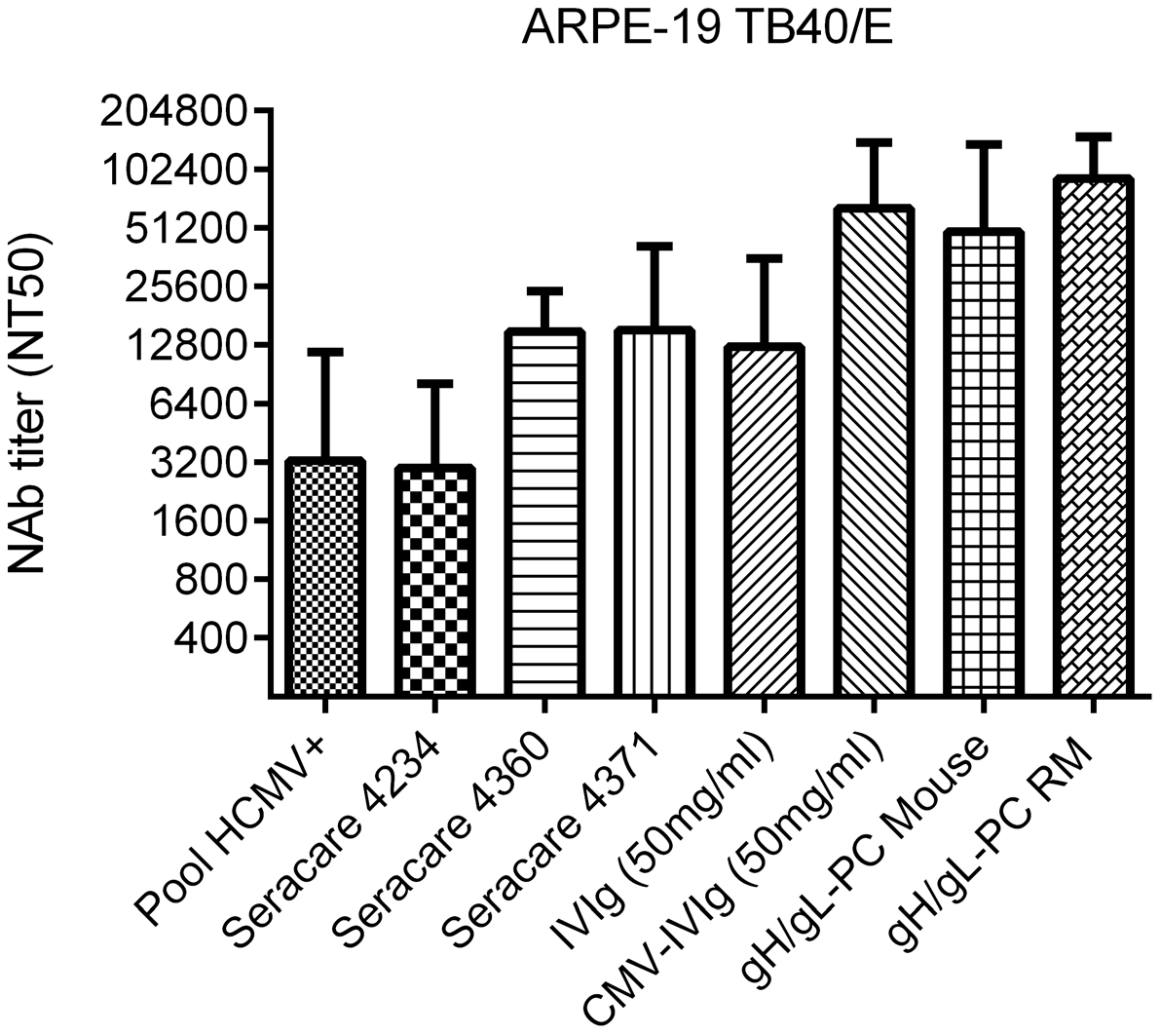
NAb in vaccinated animals and HCMV-positive human sera. NT50 titers were measured on ARPE-19 cells against TB40/E infection using commercially available HCMV IgG positive human sera (SeraCare Cat # 4234, 4360, 4371), HCMV pooled positive human sera (Pool HCMV^+^), IgG preparations (IVIg and CMV-IVIg), and in MVA-gH/gL-PC vaccinated animals after the first boost. Bars represent standard deviation of three independent experiments.

To better gauge the magnitude of the immune responses generated in vaccinated mice and RM, NAb titers were compared to a pool of HCMV seropositive sera, commercially available HCMV IgG sera, human intravenous immunoglobulin (IVIg) and CMV-IVIg preparations which all should be considered as arising from chronically infected individuals. NAb titers induced by MVA-gH/gL-PC in mice and RM markedly exceeded EC-specific NAb titers of three individual HCMV seropositive samples, a pool of seven seropositive donors (Pool HCMV+), and IVIg (Figure 7). Peak NAb titers generated in vaccinated RM and mice were similar to the titer of CMV-IVIg in sera from chronically infected humans. These titers were likely lower than immediately after primary infection. Together, these results indicate that MVA-gH/gL-PC boosts EC-specific NAb titers in two species to levels higher than those measured in pooled sera from chronically HCMV-positive humans and to comparable levels measured from concentrated hyperimmune sera (CMV-IVIg) [40], [66].

### MVA-gH/gL-PC mediated neutralization of HCMV entry into placental macrophages

**Figure 8:**
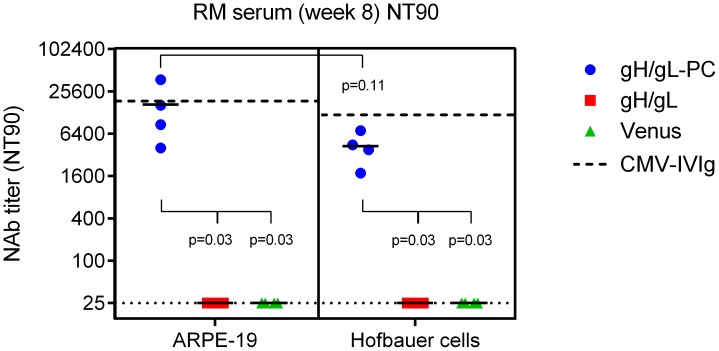
Neutralization of HCMV infection on placental macrophages (Hofbauer cells). Serum samples of RM vaccinated with MVA-gH/gL-PC, MVA-gH/gL, or MVA-Venus from week 8 after initial vaccination (Figure 6A) were used to determine the titer that prevents 90% infection (NT90) of ARPE-19 cells and HC with HCMV strain TB40/E. Dashed lines indicate the NT90 values determined for HCMV-IVIg on ARPE-19 and Hofbauer cells. Dotted lines represent the detection limit. Statistical significance was evaluated using a two-sided Wilcoxon rank-sum test.

It is hypothesized that maternal Ab plays an important role to prevent virus transmission to the fetus[67], [68], therefore we investigated neutralization of HCMV on human placental macrophages using sera from vaccinated RM described in Figure 6. Hofbauer cells (HC) are specialized placental macrophages which enter the venous circulation of the placenta and could act as a reservoir to transmit HCMV infection to the fetus [69]–[72]. We evaluated whether MVA-gH/gL-PC induces increased NAb responses to block HCMV TB40/E infection of freshly isolated HC in comparison to the standard ARPE-19 EC cell line. As shown in Figure 8 and Table S3, NAb titers induced by MVA-gH/gL-PC in RM were significantly higher than those determined from MVA-gH/gL or Venus control sera as measured in both HC and ARPE-19 cells. Yet, sera from RM vaccinated with MVA-gH/gL-PC had statistically indistinguishable NAb titers when measured on HC and ARPE-19 cells, confirming that HCMV entry into both cell types is blocked with similar potency by gH/gL-PC specific NAb (Figure 8). Thus, we have demonstrated for the first time that infection of HC by HCMV *in vitro* can be blocked with serum antibodies generated against antigen-specific targets such as gH/gL-PC. Future studies with other critical decidual and/or placental cells such as cytotrophoblasts should be attempted to conduct a fuller evaluation of placental cells whose HCMV infection is neutralized by gH/gL-PC Ab.

### MVA-gH/gL-PC vaccinated RM contain low levels of EC specific NAb in saliva

**Figure 9:**
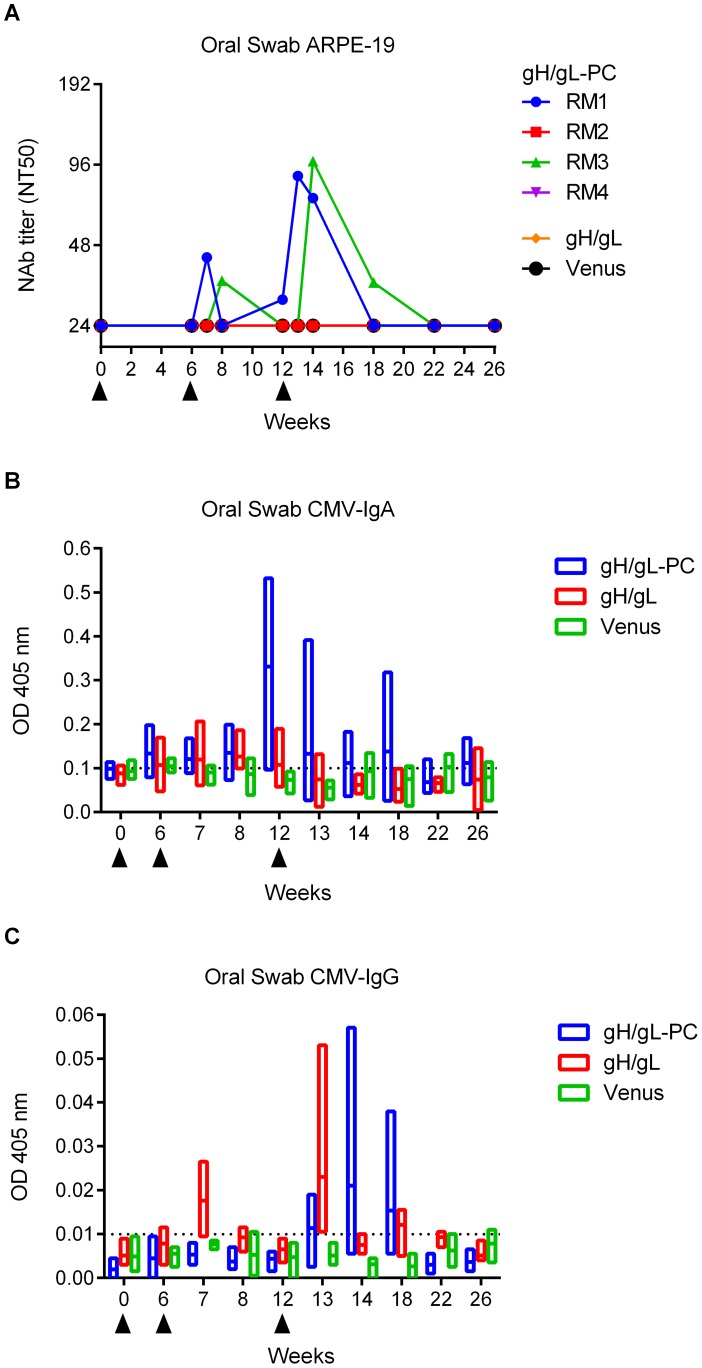
Ab response to HCMV in saliva of vaccinated RM. Oral swab samples from RM vaccinated with MVA-gH/gL-PC, MVA-gH/gL, or MVA-Venus were analyzed for presence of NAb, HCMV mucosal IgA, and HCMV-IgG. **A)** NT50 titers in saliva of individual RM measured on ARPE-19 cells against TB40/E. **B and C)** HCMV IgG and IgA levels in saliva of vaccine groups (n = 4, analysis of single animals see Sup Figure 3). Floating bar charts represent time distribution of OD values obtained using HCMV-IgA or HCMV-IgG ELISA assay. Horizontal lines represent means; bars extend from minimum to the maximum values. Dotted lines represent 99% confidence interval upper limit for mean values obtained in sera from MVA-Venus RM. Filled triangles indicate time of MVA injections.

Horizontal transmission of HCMV, including primary infection during pregnancy, is thought to occur by mucosal exposure to infectious virions. Vaccine-induced mucosal immunity may be especially relevant for limiting the potential of mucosally acquired virus from disseminating to distal sites, such as the maternal-fetal interface. We measured NAb in saliva of RM vaccinated with MVA-gH/gL-PC as a potential protective mechanism, based on the premise that oral acquisition of virus is an important route of primary infection [73]. Saliva of RM vaccinated with MVA-gH/gL-PC or MVA-gH/gL was tested for the presence of NAb that prevented HCMV TB40/E infection of ARPE-19 cells. Two of four RM vaccinated with MVA-gH/gL-PC had measurable levels of NAb in saliva following the first and second boost (Figure 9A and Table S4). In contrast to serum NAb responses induced by MVA-gH/gL-PC showing maximum NT50 levels after the first boost, saliva NAb titers were highest (NT50 of 87.0 to 99.0) after the second boost (Figure 9A and Table S4). However, saliva NAb were not maintained at peak levels and dropped gradually until they were undetectable ten weeks after the second boost. RM vaccinated with MVA-gH/gL or MVA-Venus did not have measurable NAb titers in saliva (Figure 9A and Table S4). In addition, we did not detect saliva NAb that blocked HCMV infection of MRC-5 fibroblasts in both MVA-gH/gL-PC and MVA-gH/gL vaccine groups (data not shown).

### Mucosal IgA and transudated IgG in oral saliva samples from RM

To investigate whether neutralizing activity in the oral samples of immunized RM could be attributed to transudated IgG or mucosal IgA, we evaluated total and HCMV-specific IgG and IgA levels in these samples. While total Ab levels in the oral swab samples varied over time (Figure S3), probably due to the influence of a number of variables [74], only MVA-gH/gL-PC immunized RM showed an increase in mucosal HCMV-IgA in the oral swab samples (Figure 9B). In contrast, HCMV-IgG levels in the same samples were quite low for both MVA-gH/gL-PC and MVA-gH/gL immunized RM (Figure 9C). In addition, none of the MVA-gH/gL immunized RM had measurable HCMV-specific NAb in saliva, so peaks were without interpretable significance (Figure 9A). Analysis of HCMV-IgG and HCMV-IgA Ab level variation indicates that neutralizing activity observed in RM1 oral swab samples might be attributed to mucosal IgA (Figure S3C) while that of RM3 to transudated IgG (Figure S3G). RM2 showed an interesting peak in HCMV-IgA but we could not detect any neutralizing activity in the same samples (Figure S3C). Despite the small number of animals used in this study, the results strengthen the relevance of the vaccine as a tool to induce oral secretion of IgG/IgA Ab. These results show that MVA-gH/gL-PC induces EC-specific NAb responses in saliva following vaccination of RM, which is consistent with NAb responses in saliva of HCMV-seropositive individuals [73].

### Measurement of HCMV-specific Ab avidity in sera from vaccinated mice and RM

**Figure 10:**
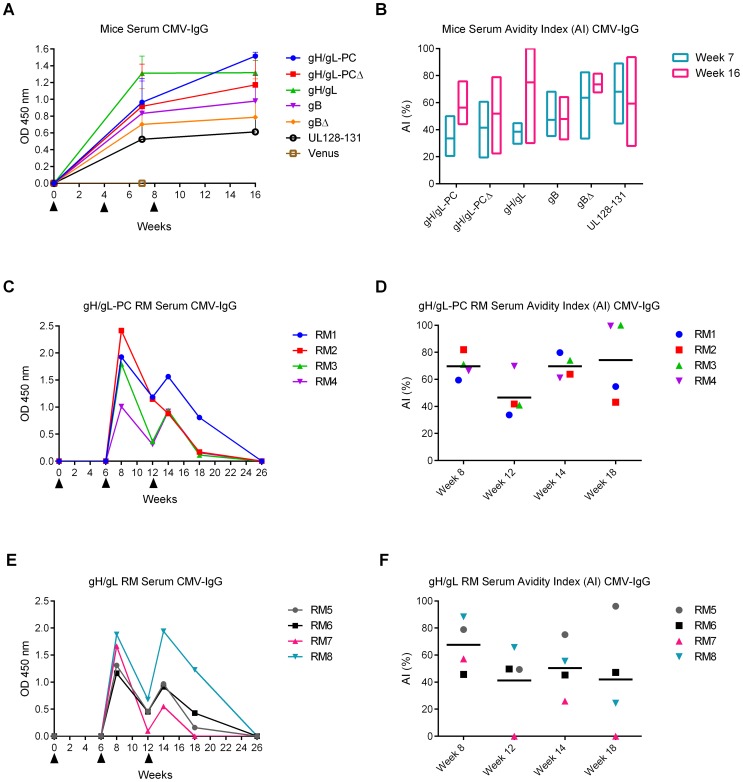
HCMV-specific Ab responses and avidity measurements in vaccinated animals. HCMV IgG levels and avidity indices (AI) in serum samples of mice and RM were determined via ELISA using HCMV particles as antigen. **A and B)** HCMV IgG levels (GMT, n = 4) and AI measured in week 7 and 16 mouse serum samples. OD values at 450 nm were normalized to week zero OD. Floating bar chart in B represents the distribution of AI in different vaccine groups; bars extend from minimum to maximum values. **C and E)** HCMV IgG levels in individual RM vaccinated with MVA-gH/gL-PC (C) or MVA-gH/gL (E) using serum samples from different time points. **D and F)** HCMV-IgG AI values in individual RM vaccinated with MVA-gH/gL-PC or MVA-gH/gL determined at indicated time points. Horizontal bars represent group means. Filled triangles in A, C and E indicate MVA vaccinations.

High avidity HCMV-specific IgG were found to be inversely correlated with transmission of HCMV to the fetus [12], [70], [75]. Therefore, we investigated avidity maturation in immunized animals using an established assay based on measurement of comparative stability of Ab:antigen complexes in the presence or absence of 6 M urea [76], [77]. With the exception of the MVA-Venus group, mice belonging to all other vaccine groups developed binding antibodies to HCMV that were stable over sixteen-weeks (Figure 10A). In contrast to animals vaccinated with MVA expressing gB, gBΔ, or UL128/130/131, all gH-related vaccine groups showed an increase in avidity index (AI) when comparing three weeks after the first boost (week seven) to the end (week sixteen) of the observation period (Figure 10B). In contrast, HCMV-specific IgG levels in RM immunized with MVA-gH/gL-PC and MVA-gH/gL were not detectable after the first vaccination, while they were present with two peaks, one week after the first and second boosts (Figure 10C and E). Total IgG Ab levels slowly decreased until they were below the detection level of the assay at the end of the observation period. Yet, at this time point, high levels of NAb preventing HCMV infection of EC were still present in the MVA-gH/gL-PC group (Figure 5B). After the first boost, both groups had an AI measurement less than 70%. Subsequently, values decreased between the two boosts and then rose to 75% in the MVA-gH/gL-PC vaccinated RM, while in RM vaccinated with MVA-gH/gL, the AI dropped to 42% (Figure 10D and F). These results indicate that MVA-gH/gL-PC induces antibodies that are simultaneously neutralizing and of high avidity, which are maintained for 6 weeks after the final boost, while the avidity of gH/gL antibodies waned in the same six week time frame.

### All MVA recombinants induce binding antibodies

We noted profound differences in the capacity of different combinations of gH/gL-PC subunits to support production of NAb. The absence or lower levels of NAb in mice vaccinated with MVA-UL128-131, gB or gH/gL in both mice and RM compared to gH/gL-PC prompted us to investigate if certain MVA recombinants were incapable of stimulating the production of BAb. Serum samples from animals vaccinated with MVA recombinants were evaluated by WB for BAb to gH/gL-PC subunits or gB. Mice vaccinated with MVA-gH/gL-PC and MVA-gH/gL-PCΔ developed BAb to gH, gL, UL128, UL131A, though not to UL130 (Figure S4). In contrast, MVA-UL128-131 not only induced BAb to UL128 and UL131A, but to UL130 as well. Mice vaccinated with MVA-gH/gL stimulated BAb to gH and gL proteins as expected (Figure S4). However, there was far greater intensity of gL and gH recognition from the gH/gL construct than MVA-gH/gL-PCΔ which may reflect steric interference with immune recognition. In addition, we detected BAb to gB in MVA-gB or MVA-gBΔ vaccinated mice (Figure S4). Interestingly, RM vaccinated with MVA-gH/gL-PC developed BAb to gH, gL, UL128, and UL130, though not to UL131A (Figure S4). As observed in mice, MVA-gH/gL elicited BAb to both gH and gL in RM, with gL showing greater immune recognition than when expressed from MVA-gH/gL-PC (Figure S4). Although all single gH/gL-PC subunits exhibited capacity for an immunological response, only their co-expression led to high titer NAb that prevented HCMV infection of EC. These results support the hypothesis that NAb against gH/gL-PC are directed against a higher order structure or conformational epitopes as a result of co-expression of five gH/gL-PC proteins.

## Discussion

It has been repeatedly confirmed that cell types besides fibroblasts, notably EC, monocytes, Langerhans cells, and decidual cells of the placenta influence HCMV natural history following initial exposure [47], [78], [79]. Moreover, antibodies that neutralize HCMV infection of non-fibroblast cell types constitute a significant proportion of total HCMV NAb [31], [40], [65]. Accordingly gH/gL-PC has become a focus of HCMV vaccine efforts since HCMV viral entry into EC and monocytes is absolutely dependent on the presence of a functional gH/gL-PC [31]–[33], [40]. However, HCMV entry into EC of different origin or modification than HUVECs or ARPE-19 cells may be gH/gL-PC independent, since in one case it has been reported that the gH/gL-PC defective HCMV strain Towne is apparently able to infect and replicate in fibroblasts, microvasculature endothelial cells, and immortalized retinal epithelial cells with comparable efficiency [80]. An obstacle for targeting gH/gL-PC by vaccination is that formation of EC-specific NAb requires co-expression of all five gH/gL-PC subunits. This requires that vaccines targeting gH/gL-PC-neutralizing epitopes co-express all the individual proteins, to ensure cell surface expression of multimeric protein structures that are needed to form conformational epitopes [41].

Our previous study provided a template for efficiently stimulating HCMV gH/gL-PC-specific NAb in vaccinated RM. BAC-derived MVA was engineered to co-express all five RhCMV gH/gL-PC proteins, and RM immunized with MVA-RhgH/gL-PC developed antibodies that neutralized EC infection with a virulent RhCMV strain[46]. One advantage of this approach is using bacterial genetics to serially introduce the five genes encoding gH/gL-PC proteins, which shortens the time for vector construction. In addition, a single vector for co-expression means that every vaccine infected cell *in vivo* can express all combinations of multimeric protein structures that encode epitopes stimulating NAb. This study extends our work in RhCMV with a focus on MVA vectors that express two forms of HCMV gH/gL-PC. We show that both forms of gH/gL-PC stimulate potent serum Ab titers in murine and primate hosts that neutralize infection of EC, HC and fibroblasts with different HCMV strains.

Our biochemical studies provided valuable insight into the role of gH as a membrane anchor and the Ab accessible surfaces of the complex. The importance of the contribution of gH/gL towards NAb stimulation by gH/gL-PC is emphasized, because expression of UL128-130-131A in the absence of gH/gL fails to stimulate EC-NAb. Using co-IP, in which a mAb to gH or polyclonal antisera to UL130 results in the pull-down of all five gH/gL-PC proteins is consistent with the formation of a complex composed of five subunits. This suggests that both gH and UL130 are accessible by Ab in the secreted and cellular forms of the gH/gL-PC (Figures 3A-D). The FC results provide further evidence, but do not prove that the TM-containing gH/gL-PC is accessible by gH and UL130 Ab on the cell surface (Figure 4). It is significant that we detected a cell-associated trimolecular UL128-UL130-UL131A complex using co-IP after in *vitro* cell infection with MVA-UL128-131; since vaccination with MVA-UL128-131 did not cause the development of NAb preventing HCMV infection of the cell types we investigated. Paradoxically, UL128-UL130-UL131A proteins form a sufficiently stable intracellular complex to be detected by co-IP using the UL130 polyclonal antisera and BAb were stimulated for each of the UL128-131A subunits. Moreover UL130 is detectable by FC on the surface of cells infected with MVA-UL128-131. These data provide evidence that the gH/gL scaffold facilitates the production of NAb, and that complexes of UL128-130-131A proteins without gH/gL are insufficient to induce NAb. MVA expressing only gH/gL stimulated EC-specific NAb following vaccination in both mice and RM. However, the titers generated with MVA-gH/gL were one to two orders of magnitude lower than those generated with either MVA-gH/gL-PC or MVA-gH/gL-PCΔ. We conclude that gH/gL-PC composed of five proteins results in higher titer NAb than elicited by gH/gL complexes. Our results demonstrate that co-expression of all five subunits of gH/gL-PC is an efficient mechanism to stimulate high titer EC-specific NAb. However, alternative explanations could account for our observations, since the stoichiometry of the subunits is unknown. For example, gH might form multiple complexes with different combinations of the other gH/gL-PC proteins which collectively would co-precipitate with gH.

MVA-gH/gL-PC and MVA-gH/gL-PCΔ were generated to evaluate the impact of the TM domain on the localization of the complexes, either within the cell or secreted in the medium, and whether membrane-attached gH/gL-PC with full-length gH or soluble gH/gL-PCΔ with TM-deleted gH is a better immunogen to induce NAb [53], [81]. Using a WB approach, we compared efficiency of expression of gH/gL-PC subunits in the whole cell when gH was expressed with or without a TM domain and found no difference in the expression levels of individual gH/gL-PC subunits. We conclude from our results that the presence or absence of the TM domain determines if the complex will be secreted into the medium. Co-IP was used again to discover that the gH/gL-PCΔ remains intact in the medium, as we were able to account for all five subunits using the mAb to gH and a polyclonal sera against UL130. This finding helps explain why we found strong NAb responses when vaccinating with MVA-gH/gL-PCΔ, since the secreted complex remains intact, and likely stimulates the generation of the required conformational Ab that we and others find to be highly neutralizing against EC and fibroblast infection [41]. We found a similar result with gB, with or without the TM domain. In that case, in the presence of the TM domain, both the precursor and membrane-spanning processed forms are detected intracellularly, although not in the medium. In contrast, in the case of the expression product without the TM, we found that the processed C-terminal fragment is expressed both intracellularly and in the medium. A further independent confirmation of the influence of the TM on the localization of complexes was carried out using FC or IF. The TM domain localized the protein to the cell surface, presumably co-localized with other gH/gL-PC subunits, while the absence of the TM dramatically reduced cell-surface expression. These results agree with prior studies by Ryckman and collaborators investigating the gH/gL-PC and others who investigated gB secretion requirements [39], [53], [82], [83].

Our results indicate that MVA-gH/gL-PC and MVA-gH/gL-PCΔ boost the immune system to produce comparable NAb titers. However, induction of maximum NAb responses required only one boost with MVA-gH/gL-PC versus two boosts with MVA-gH/gL-PCΔ. The necessity for only two vaccinations with MVA-gH/gL-PC to induce maximum NAb titers could reduce anti-vector immunity and simplify the regimen. The lower number of vaccinations using MVA-gH/gL-PC and faster kinetics to obtain a maximal NAb response favors that form of the construct for clinical translation and provided the rationale why it was chosen instead of MVA-gH/gL-PCΔ for evaluation in monkeys. However, further experiments including long-term studies are required to conclude if gH/gL-PC with full-length gH is superior to TM-deleted gH for induction of durable and potent NAb. In the case of gB, we anticipated that gBΔ should induce far greater NAb titers than full-length gB on MRC-5 fibroblasts based on work by Plotkin and associates and our prior work [53], [81], [84]. While the results with fibroblasts and different forms of gB were unexpected, there were significant differences in the experimental design of our previous studies as well as those from the Plotkin laboratory. For instance, the clinical-like strain TB40/E was not previous employed and the current vaccine virus was derived from an MVA-BAC as opposed to the cell-culture method that we used in our previous study.

Our studies contribute to the understanding of the role of gH/gL-PC as a pivotal target of the host NAb response to HCMV [33], [45]. We demonstrated that NAb induced by MVA-gH/gL-PC that prevent HCMV infection of EC are at higher titers when compared to NAb that prevent HCMV infection of fibroblasts. This is consistent with NAb responses observed after natural HCMV infection [31], [40]. The strength and utility of MVA-gH/gL-PC as a vaccine is illustrated by its ability to boost the immune system in two different animal species to reach peak levels of serum NAb that are higher than those measured from IVIg and roughly equivalent to CMV-IVIg. The significance of equaling or surpassing NAb levels in IVIg and CMV-IVIg is to develop an approach that supersedes the efficacy of these products that failed to provide adequate protection against congenital HCMV infection and disease in the recently reported randomized Phase 2 trial [12], [13]. Noteworthy are studies with HC, a placental cell of fetal origin and depot of HCMV that may contribute to transplacental crossing of HCMV, a potential initiating factor of congenital infection and disease [5], [78]. We observed strong neutralization of the clinical-like isolate TB40/E which makes the vaccine a possible means to prevent HCMV infection of the placenta and/or fetus. Studies of RhCMV infection of pregnant RM provide an excellent model to investigate the capacity of a rhesus gH/gL-PC vaccine to limit congenital infection as we described [85].

We discovered that the MVA-gH/gL-PC vaccine stimulated EC-specific NAb in saliva of vaccinated RM. Furthermore, we characterized the Ab class that was found in the saliva as either mucosal IgA or transudated IgG. Interestingly, the peaks of both IgA and IgG corresponded to peak NAb titers in two of the four RM. As anticipated, MVA-gH/gL-PC vaccinated animals showed more peaks of IgA and IgG than MVA-gH/gL vaccinated animals. Recently, low level saliva NAb to HCMV were measured in HCMV-positive individuals, so it is relevant that we measured comparable NAb after MVA-gH/gL-PC vaccination [73]. While we could not measure saliva IgA, IgG, or NAb in all RM, this could be attributable to genetic diversity of the RM we studied, analogous to findings that not all HCMV-positive humans had measurable saliva NAb. Moreover, we cannot exclude that some RM developed saliva NAb titers of biological relevance that were under the detection limit of the assay. We conclude that MVA-gH/gL-PC induces NAb in saliva from RM analogous to naturally infected humans [45], [73].

The apparent paradox that MVA-gH/gL-PC induced NAb that prevented HCMV infection of fibroblasts can be explained by proposing that a subset of the NAb repertoire generated by MVA-gH/gL-PC are uniquely directed against gH/gL epitopes without the accompanying UL128-UL130-UL131A subunit contribution [41]. It is striking that NAb responses induced by MVA-gH/gL-PC and MVA-gH/gL that block HCMV infection of fibroblasts are almost identical to each other in titer and kinetics. This suggests either that gH/gL subunits as part of the gH/gL-PC encode neutralizing epitopes similar to those presented by the gH/gL heterodimer, or that MVA-gH/gL-PC generates both gH/gL heterodimers as well as the gH/gL-PC that is composed of all five proteins, with each complex stimulating NAb. Further biochemical studies will clarify which complexes are generated by the MVA-gH/gL-PC vaccine. The results of this study indicate that gH/gL-PC can be used to stimulate NAb against both the fusion and endocytic pathways of virus attachment and entry. An important contribution highlights that the gH/gL-PC is dispensable for Langerhans-type dendritic cells [79]. Nevertheless, the property of the MVA-gH/gL-PC vaccine to neutralize both fibroblast and EC infection by HCMV, suggests that it would be important to test if the MVA-gH/gL-PC vaccine will generate NAb that will be effective on Langerhans-type dendritic cells. A recent study has reported on non-infectious particles generated from HCMV strain Towne that likely express a subset of entry glycoprotein complexes such as gB, gH/gL, gM/gN but unlikely intact gH/gL-PC due to a defective UL128-UL131A locus [86]. As expected due to the missing gH/gL-PC and consistent with our data, they found equivalent levels of NAb measured on fibroblast or ARPE-19 cells. Comparison with results from our study showed that while neutralization on MRC-5 fibroblasts was comparable, levels of NAb that neutralize infection of ARPE-19 cells were far lower using the DB preparation than what we measured using the MVA-gH/gL-PC construct by two orders of magnitude, further confirming that the UL128-UL131A proteins are required in combination with gH/gL to induce high titer EC specific NAb. These results suggest a qualitative difference of DB and MVA-gH/gL-PC in eliciting NAb that prevent HCMV infection of EC.

The implications of our work on the gH/gL-PC can be appreciated as a comparable model to the main HIV envelope glycoprotein complex. HIV research has shown that vaccine constructs which resemble the antigenic structure of the native trimeric gp120/gp41 (gp140) envelope spike induce more potent NAb than those using only gp120 monomers [87]. Our results complement these findings by showing in mice and RM that a vaccine approach based on all five gH/gL-PC proteins is overwhelmingly more effective for high titer NAb induction than using only protein subsets of the complex. Our findings together with a recent report from Novartis that demonstrates similar vaccine properties of the gH/gL-PC using an alphavirus approach will influence virus vaccine design to optimize the generation of potent NAb responses by co-expressing multimeric subunits in the same vector construct [14], [88], [89].

The strict human host specificity of HCMV precludes evaluation of protective efficacy by MVA-gH/gL-PC in any currently used animal model such as RM. In addition, although the genomes of HCMV and RhCMV are largely co-linear, the two viruses likely do not share cross-reactive immune responses, excluding RhCMV as a challenge virus to analyze protection by HCMV specific immunity in RM [90], [91]. We confirmed that sera of DNA-typed RhCMV-positive RM contain no cross-reactive NAb that prevent HCMV infection of EC and fibroblasts, although these same sera neutralize RhCMV infection of primary rhesus kidney epithelial cells (data not shown). Consequently, protective efficacy by MVA-gH/gL-PC or any other vaccine against HCMV can only be evaluated in humans. Our prior work using MVA as a vaccine vector in which RhCMV-negative RM were partially protected from acquiring a virulent RhCMV demonstrates the suitability of this vector system as a candidate prophylactic vaccine in a relevant herpesvirus model [92]. In that study and our more recent study involving the rhesus gH/gL-PC only two administrations of vaccine were required to attain partial protection [46]. MVA as a vaccine vector is well established and is in the clinic (NCT01941056) as a therapeutic vaccine to control HCMV viremia in stem cell transplant recipients and to prevent HIV infection [93], [94].

Our approach of measuring NAb in a primate species after MVA vaccination comes closest to actual human vaccination without requiring FDA approval. These studies lay the groundwork for a step-wise clinical evaluation of the MVA-gH/gL-PC, either as a single agent or combined with other prominent NAb or cellular immune targets in HCMV-negative healthy adults, pregnant women, and adolescents, especially those exposed to HCMV in a daycare setting [95], [96]. While it is unknown if any HCMV vaccine will prevent reinfection by a second strain of the virus, our approach has been developed to prevent primary infection [97]. It will require clinical investigation to assess if a gH/gL-PC containing vaccine is a successful therapeutic option, either alone or combined with other vaccine antigens.

In conclusion, we have generated an MVA vaccine vector expressing five gH/gL-PC subunits that elicited high titer EC and fibroblast specific NAb in mice and RM. This study will influence HCMV vaccine design for the following reasons. 1) It outlines a strategy to effectively prevent the infection of EC and fibroblasts, primary portals for HCMV replication. 2) HCMV infection is neutralized on placental macrophages or HC, a known HCMV depot in the placenta. 3) It introduces a vector system to assemble all five gH/gL-PC subunits, a requirement for the formation of conformational neutralizing epitopes for *in vivo* applications. 4) It is the first vaccine strategy that targets neutralizing epitopes formed by the full complement of HCMV gH/gL-PC subunits expressed from a translational vaccine vector platform with an extensive clinical safety record. 5) The strategy is equally effective to elicit high titer NAb in mice and RM. 6) NAb in mice and RM induced by MVA-gH/gL-PC exceed those of HCMV positive serum and IVIg preparations. 7) The vaccine strategy induced NAb preventing infection of EC with titers that exceed those induced by gH/gL or gB. The pivotal test is to vaccinate HCMV-negative adolescents with MVA-gH/gL-PC with or without additional antigens and assess efficacy in preventing infection and shedding. Equally important is preventing infection in pregnancy and viral transmission to a fetus. If successful, a gH/gL-PC vaccine may reduce the morbidity and burden of congenital HCMV as well as in immunosuppressed hosts from primary or reactivated infection.

## Materials and Methods

### Ethics statement

#### Human placenta

With written informed consent, term (>37 weeks gestation) placentas from HIV-1 and Hepatitis B seronegative women (>18 years of age) were obtained immediately following elective caesarian section without labor from Emory Midtown Hospital in Atlanta, GA. Approval of the study was granted from the Emory University Institutional Review Board (IRB). Written informed consent was obtained from donors, and samples were de-identified prior to handling by laboratory personnel.

#### Rhesus Macaques

The animal protocol (#17158) assigned to this study was approved prior to implementation by the Institutional Animal Care and Use Committee (IACUC) at UCDavis, and all procedures conformed to the requirements of the Animal Welfare Act. This study was carried out in strict accordance with the recommendations in the Guide for the Care and Use of Laboratory Animals of the National Institutes of Health and in accordance with the recommendations of the Weatherall report, “The use of nonhuman primates in research.” Activities related to animal care including housing, feeding, and environmental enrichment were performed in accordance with Institutional Animal Care and Use Committee (IACUC)-approved standard operating procedures (SOPs) at the California National Primate Research Center (http://www.cnprc.ucdavis.edu). Animals were anesthetized during blood collection, immunization, and viral challenges. The state of the anesthetized animals was constantly monitored (movement, respiratory rate, etc.). Analgesics and veterinary care were provided, as needed, based on the recommendation of the attending veterinarian. All of the studies were non-terminal, and all animals were returned to the CNPRC general colony at the completion of the study.

#### Mice

The IACUC of the Beckman Research Institute of City of Hope approved protocol #98004 assigned for this study. All study procedures were carried out in strict accordance with the recommendations in the “Guide for the Care and Use of Laboratory Animals of the National Institutes of Health.” Animals were anesthetized for MVA infections and visually monitored by the investigator for recovery from anesthesia until the animal was supine and ambulatory. Animals were observed daily by trained animal care staff, and animals requiring care were referred to the attending veterinarian for immediate care and euthanasia, if necessary. Analgesics were provided, as needed, at the recommendation of the attending veterinarian. Animals had longitudinal blood draws following MVA immunizations, and all animals will be terminated according to the *Guide for the Care and Use of Laboratory Animals* and PHS Policy on the Humane Care and Use of Laboratory Animals. Methods of euthanasia followed the most current “Report of the AVMA Panel on Euthanasia” (http://www.avma.org/kb/policies/documents/euthanasia.pdf). Methods were chosen to minimize pain and distress to the mice. Mice were terminated using a CO_2_ inhalation chamber.

### Viruses and cells

All MVA recombinants, except MVA expressing the fluorescent marker Venus, were reconstituted from MVA-BAC as described below [46]. The generation of MVA-Venus has been described elsewhere [98]. MVA recombinants were propagated on BHK and CEF cells as described previously [58]. CEF cells (Charles River Laboratory, Wilmington, MA, USA) were maintained in serum-free medium (VP-SFM, Invitrogen, Carlsbad, CA, USA). To generate MVA stocks for *in vitro* and *in vivo* experiments, MVA was prepared from infected BHK cells, purified once by 36% sucrose density ultracentrifugation, and resuspended in 1 mM Tris-HCl (pH 9)[53], [92]. MVA stocks were maintained at −80°C.

HCMV strain TB40/E-GFP (kindly provided by Christian Sinzger, Ulm University, Germany)[49], [99] was reconstituted from BAC-DNA in MRC-5 fibroblasts. HCMV strain TR-GFP was a gift from Jay Nelson (Oregon Health & Sciences University, Portland, OR, USA). The isolation of clinical-like HCMV strain VHL/e has been described elsewhere [63]. ARPE-19 and MRC-5 cells were purchased from ATCC and maintained in Dulbecco's minimal essential medium (DMEM, Corning, Corning, NY, USA) or minimal essential medium (MEM, Corning), respectively, supplemented with 10% fetal bovine serum (FBS, Hyclone, Logan, UT, USA). HUVECs were maintained in VascuLife Basal Medium added with VascuLife EnGS LifeFactors (all from Lifeline Cell Technology, Frederick, MD, USA). HCMV stocks were generated following virus propagation in ARPE-19 cells. Cells were monitored for cytopathic effects (CPE) and re-seeded at a dilution of 1∶2 until 70-80% of the ARPE-19 cells were infected. Cells were again re-seeded at a dilution of 1∶2 and grown for additional three to four days. Virus particles were concentrated from clarified medium by ultracentrifugation (70000×g for one hour) over 20% sucrose (w/v) in Tris-buffered saline (0.1 M Tris-Cl, pH 7.4, 0.1 M NaCl). Concentrated virus was resuspended in Tris-buffered saline and stored at −80°C. Virus stocks were titrated on each cell type (ARPE-19, HUVEC, MRC-5, HC) by immediate-early-1 (IE1) immunostaining to use approximate the same amounts of plaque forming units (PFU) for the different cell types in the neutralization assay.

### Isolation and culture of HC

In order to isolate HCs, the decidua basalis was dissected from the placenta, as previously described [100]. The tissue was thoroughly washed in Hank's balanced salt solution (HBSS) to minimize peripheral blood contamination and mechanically dispersed in complete medium (RPMI supplemented with 10% FBS, 1 mM L-glutamine, and 1% pen/strep). The minced tissue was re-suspended in complete medium containing 10% Trypsin/EDTA (Sigma-Aldrich, St. Louis, MO, USA) for 1 hour, followed by resuspension in media containing 1 mg/ml collagenase IV (Sigma-Aldrich), 10 U/ml dispase (Worthington Biochemical Corp., Lakewood, NJ, USA), and 0.2 mg/ml of DNAse I (Sigma-Aldrich) and incubated in a shaking water bath at 37°C for 1 hour. The digested tissue was washed with PBS and passed through a 70 µm cell strainer (BD-Falcon Biosciences, Lexington, TN, USA). The mononuclear cell population was isolated by density gradient centrifugation on Histopaque-1077 (Sigma-Aldrich). CD14^+^ Magnetic Cell Sorting was performed using anti-CD14 magnetic beads (Miltenyi Biotech, Bergisch Gladbach, Germany) as recommended by the manufacturer. The purity of the HC population was assessed by CD14 staining and was on average greater than 97%.

### Transfer vector construction

Transfer vectors for gene insertion into the Deletion II site (Del2) [101], intergenic region 3 (IGR3) [102], and the insertion site between the essential ORFs G1L and I8R [52] of the MVA-BAC [60], [103] were generated as follows (Figure 1A). Approximately 700 bp on either side of the MVA insertion site were PCR amplified from MVA-BAC (Table S5: primer pairs P1/2 and P3/4 for Del2; P5/6 and P7/8 for IGR3; P9/10 and P11/12 for the G1L/I8R sites) and cloned via *SalI* or *SpeI* into the *XhoI* or *XbaI* restriction sites, respectively, between the two *I-CeuI* recognition sequences of plasmid pCeu2 [104]. Then, the kanamycin resistance gene (Kan^R^) *aphAI* and the homing endonuclease *I-SceI* recognition sequence of plasmid pEPkan-S2 [105] were PCR amplified with primers that provided a 50 bp duplication of the MVA insertion site (Table S5: P13/14 for Del2; P15/16 for IGR3; P17/18 for G1L/I8R). The amplified fragments were introduced via *SalI* into the *XhoI* restriction site between the cloned 700 bp MVA sequence flanks. Next, the mH5 promoter of plasmid pZero2-mH5 [58] was PCR amplified with primers that encoded a multiple cloning site (*MCS*) (Table S5: P19/20) and cloned via *SpeI* into the *AvrII* site between the left 700 bp MVA sequence flank and the *aphAI-I-SceI* cassette (Figure 1A). While the mH5 and *MCS* sequences were introduced in the sense orientation between the 700 bp G1L/I8R MVA sequence flanks, they were cloned in the antisense orientation between the Del2 and IGR3 700 bp sequence flanks (Figure 1A). The final constructs contained an *MCS* downstream of the mH5 promoter, an *aphAI-I-SceI* cassette flanked by a 50 bp duplication, and two 700 bp MVA sequence flanks at both ends of the transfer constructs (Figure 1A). The resulting plasmids for gene transfer into Del2, IGR3, or G1L/I8R, were named pEP-Del2, pEP-IGR3, or pEP-G1L, respectively. These plasmids allow easy cloning into the *MCS* and release of the transfer construct via digestion with *I-CeuI, PmlI*, or other restriction sites on the 700 bp MVA sequence flanks. All final cloned sequences were confirmed by sequencing. Sequence files for all transfer vectors are available upon request.

### Cloning of HCMV genes

HCMV genes were derived from TB40/E-BAC4 (Accession# EF999921) [49] and cloned as transfer construct for *en passant* mutagenesis [60], [103]. Genes were cloned via added *PmeI* and *AscI* restriction sites into *EcoRV* and *AscI* restriction sites of the *MCS* of the corresponding transfer plasmids. All ORFs were also cloned with the Kozak sequence GCCGCCACC directly preceding the ATG start codon. For gene transfer of UL128 or UL131A, synthesized intron-less coding sequences (GenScript, Piscataway, NJ, USA) were cloned into pEP-Del2 or pEP-IGR3 (Figure 1A), resulting in pEP-Del2-UL128 or pEP-IGR3-UL131A, respectively. To generate transfer constructs for gH, either as full-length or TM-deleted coding sequence with C-terminal myc-tag epitope, the gH ORF was PCR amplified (Table S5: P29/30 or 31/32) from TB40/E-BAC4 and cloned into pEP-G1L (Figure 1A). The C-terminal deletion of gH, removing both the TM and cytoplasmic domains, corresponded to aa 719-743. The derived plasmids were termed pEP-G1L-gH or pEP-G1L-gHΔTM, respectively. For insertion of full-length gB or gBΔ (Figure 1C)[53], PCR fragments (Table S5: P33/34 and P35/36) amplified from TB40/E-BAC4 were cloned into the pEP-G1L plasmid, resulting in pEP-G1L-gB and pEP-G1L-gBΔ, respectively. The C-terminal deletion of gBΔ spanned aa 682 to 907 of the full-length gB protein similar as described [53]. In contrast, the recombinant gBΔ protein of the recent clinical phase II trial was derived from HCMV strain Towne (Accession #M22343)[106]. This gBΔ protein is a fusion protein of 807 aa with mutagenized protease cleavage site that was generated by an internal deletion of the TM domain between aa 715 and 772 of the full-length gB protein [106]. Towne and TB40/E gB proteins are 99% identical with a total of seven aa difference over the entire length of the 907 aa protein. Transfer constructs for UL130 and gL were generated as follows. The mH5 promoter of plasmid pZero2-mH5 [58] was PCR amplified (Table S5: P19/20) and cloned via the pGEM-T vector system (Promega, Madison, WI, USA), resulting in pGEM-T-mH5. Then, UL130 or gL was PCR amplified from TB40/E-BAC4 [49] (Table S5: 21/22 or P23/24) and cloned (via *PmeI* and *AscI* into the *EcoRV* and *AscI* restriction sites) into pGEM-T-mH5. The *aphAI-I-SceI* cassette of plasmid pEPkan-S2 [103] was then PCR amplified (Table S5: P25/26 or P27/28) and introduced via the *KpnI* (UL130) or *MfeI* (gL) restriction sites within the cloned ORFs, resulting in pEP-UL130 or pEP-gL, respectively. The final constructs contained the gene sequence with upstream mH5 promoter and an internal *aphAI-I-SceI* cassette flanked by a 50 bp gene duplication of the cloned ORFs [46]. All cloned sequences were confirmed by sequence analysis.

### *En passant* mutagenesis

Vaccinia virus expression cassettes for each HCMV gene were recombined into the MVA-BAC by *En passant* mutagenesis in *E. coli* strain GS1783 as described [103]. For insertion of UL128, UL131A, gH, or gB sequences, the corresponding transfer constructs (pEP-Del2-UL128, pEP-IGR3-UL131A, pEP-G1L-gH, or pEP-G1L-gHΔTM, pEP-G1L-gBΔ, pEP-G1L-gB) were released via *I-CeuI* and introduced into MVA insertion sites by initial Red recombination (Figure 1A). After selection of kanamycin resistant recombinants and verification of BAC constructs by colony-PCR and restriction fragment length analysis (RFLA), the selection marker was excised by expression of *I-SceI* to introduce a double strand-break at the *I-SceI* recognition site followed by a second Red recombination between the 50 bp duplications of the MVA genome flanking the Kan^R^ selection marker (Figure 1A). Recombinants were screened by colony PCR and RFLA. The correct insertion of each gene was confirmed by sequencing. To insert UL130 or gL into the MVA-BAC, the cloned gene sequence with internal *aphAI*-*I-SceI* cassette flanked by a 50 bp gene duplication and upstream mH5 promoter was amplified via PCR (Table S5: P37/38 or P39/40), and used for *En passant* mutagenesis as described [46]. UL130 and gL were inserted at the left and right BAC vector as shown in Figure 1B. Transfer vectors as shown for Del2, IGR3, or G1L/I8R in Figure 1A were not generated for these positions, since they are uncommon MVA insertion sites (Figure 1A and 1B). The first Red recombination was performed by 50 bp extensions provided by the 5′ ends of the primers used to amplify the entire insertion construct (Table S5). The resistance marker was then excised by a second Red recombination as described above (Figure 1A) [46].

### Virus reconstitution

Virus reconstitution from BAC-DNA was performed on BHK cells in the presence of helper Fowl pox virus FPV HP1.441 (FPV) [107] (provided by Bernard Moss, NIAID) similar to published procedures [46], [60], [108]. Briefly, BAC-DNA was prepared from *E. coli* by alkaline lysis [109]. ∼2–4 µg purified BAC DNA was transfected into BHK cells using X-tremeGENE HP DNA transfection reagent (Roche, Mannheim, Germany) according to manufacturer's instructions. BHK cells were seeded 16–20 h before transfection at 1×10^4^ cells/cm^2^. Four hours post-transfection, BHK cells were infected with FPV at multiplicity of infection (MOI) of 0.1 to initiate virus transcription [46]. Reconstitution was monitored by expression of GFP from BAC vector constructs [46].

### Antibodies

Rabbit polyclonal antiserum against GST-tagged UL128 was purchased from ATCC [33]. Anti-HCMV gH mAb 11-1-1 or 14-4b [59], anti-HCMV gB mAb p7-17 [20], and anti-HCMV IE1 mAb p63-27 [110] were kindly provided by W. J. Britt (University of Alabama, Birmingham, AL, USA). Rabbit polyclonal antisera to HCMV UL130, UL131A, and gL were raised against the following peptide sequences: CRMPRTASKPSDGNV (1) and PWSTLTANQNPSPLWSKLTYC (2) were used to generate UL130 antisera. The antiserum raised against the first peptide sequence (1) was used for Immunoblot analysis; and the antiserum raised against the second UL130 peptide sequence (2) was used for co-IP. QCQRETAEKNDYYRC was used to generate the UL131 antiserum. Peptide sequence CKQTRVNLPAHSRYGPQAVDAR was used to derive the gL antiserum (GenScript).

### Immunoblot

Immunoblot to confirm HCMV gene expression from MVA was performed similar to published standard protocols [46], [53]. Briefly, BHK cells (80–90% confluent) were infected with the MVA recombinants at MOI of 5 and grown for 16–20 h. Infected cells were harvested, centrifuged at 300×g, and lysed directly by resuspension in SDS sample buffer (2% SDS, 100 mM dithiothreitol [DTT], and 125 mM Tris-HCl [pH 8.8]). Proteins were boiled, electrophoretically separated by SDS-PAGE, and blotted to a PVDF membrane. Hybridoma supernatant or ascites fluid of anti-HCMV gH mAb 11-1-1 were used at dilutions of 1/10 or 1/1000, respectively. UL128, UL130, UL131A, and gL were detected with rabbit polyclonal antisera at dilutions of 1/3000 to 1/5000. gB proteins were detected with hybridoma supernatant of anti-gB mAb p7-17 at dilution of 1/10. Proteins were visualized with secondary antibodies; anti-rabbit or anti-mouse IgG Ab coupled to horseradish peroxidase (HRP), followed by chemiluminescence detection (Pierce ECL WB substrate, Pierce, Rockford, IL, USA). To detect secreted proteins, confluent CEF cells (80-90%) were infected with the MVA recombinants (MOI of 5) and grown in serum free medium (VP-SFM; Invitrogen) for 16–20 h. The medium was harvested, clarified by centrifugation at 1000×g, and 20-fold concentrated over Amicon Ultra 4 centrifugal filters (Millipore, Billerica, MA, USA). The concentrate was mixed with SDS samples buffer. Prepared medium samples and cell lysates of the infected CEF were analyzed by WB as described above.

BAb in serum samples from mice and monkeys were detected via Immunblot as follows. Confluent ARPE-19 cells were infected with different combinations of replication-deficient Ad vectors kindly provided by David Johnson (Oregon Health & Sciences University, Portland, OR, USA)[39]. Cells were co-infected with 20 MOI of Ad expressing the tetracycline (tet)-transactivator (Ad tet-trans), gH and gL, with Ad tet-trans and UL128, UL130 or UL131A, with Ad tet-trans and gB, or with Ad tet-trans alone as a control. Infected cells were harvested four days post infection, centrifuged at 300×g, resuspended Tris-HCl buffer (10 mM Tris-HCl pH 7.9, 1.0 mM EDTA, and Complete Mini protease inhibitor cocktail tablets (Roche)), and sonicated. Samples were lysed in SDS buffer, boiled, and used for SDS-PAGE. Proteins were separate on 8% (gH, gL and gB) or 12% (UL128, UL130 and UL131A) SDS-polyacrylamide gels using MOPS SDS running buffer (50 mM MOPS, 50 mM Tris-base, 0,1% SDS, 1.0 mM EDTA, pH 7.7), transferred to a PVDF membrane, and blocked over night with PBS-Tween 0.1% (PBS-T)/6% BSA. Inactivated serum from immunized animals was used as a primary Ab at a dilution of 1/15000 in PBS-T/5% BSA. Secondary anti-mouse HRP conjugate (Promega) and anti-monkey HRP conjugate (KPL, Gaithersburg, MD, USA) were employed in a dilution of 1/20000 in PBS-T/2.5% BSA. Pierce ECL WB substrate was used for chemiluminescence detection.

### Immunoprecipitation

Immunoprecipitation (IP) was performed as follows. BHK cells (80–90% confluent) were infected with MVA recombinants at MOI of 5 and harvested after 16–20 h of incubation. All following steps were performed at 4°C or on ice. Cells were lysed in buffer containing 0.5% (w/v) Triton X-100, 100 mM Tris-HCl (pH 8.0), 100 mM NaCl, and Complete Mini protease inhibitor cocktail tablets (Roche). Lysates were cleared by centrifugation at 10000×g and incubated two times for 1 h with recombinant Protein G Agarose beads (Invitrogen) to remove nonspecific binding. IP was performed by incubation of pre-cleared lysates for 2 h to over night with recombinant Protein G Agarose beads coupled to mouse anti-gH mAb 11-1-1 or UL130 polyclonal antibodies (UL130 antiserum). As a control for anti-HCMV gH 11-1-1 mAb or UL130 antiserum, samples were incubated with beads either coupled to a mouse IgG control Ab (Biolegend, San Diego, CA, USA) or rabbit UL130 pre-immune serum, respectively. The beads were washed six times with PBS and boiled in SDS sample buffer. Immunoprecipitated proteins were detected by immunoblot as described above. For immunoprecipitation of secreted proteins, CEF cells grown in VP-SFM were infected with 5 MOI of MVA-gH/gL-PCΔ and incubated for 16–20 h. The medium was harvested, clarified by centrifugation at 1000×g, and 6-fold concentrated via Amicon Ultra 4 centrifugal filters (Millipore). Samples were subjected to IP and WB as described. UL130 and gL after co-IP with rabbit UL130 polyclonal antibodies were detected via Immunoblot with Clean Blot IP detection reagent (Pierce) as secondary Ab to reduce the detection signal of the heavy and light chain of the immunoprecipitating Ab.

### Flow cytometry (FC)

For FC detection of gH and UL130 cell surface expression, confluent BHK cells were infected at an MOI of 5 with MVA recombinants. Cells were harvested 8 hours post-infection and washed twice by centrifugation at 300×g and resuspension in FC-buffer (PBS, 0.1% BSA). Cells were incubated with primary and secondary Ab diluted in FC-buffer for 30 minutes at 4°C. Hybridoma supernatants of the primary Ab anti-HCMV-gH mAb 14-4b were used at dilution of 1/10, while UL130 rabbit antiserum was used at a dilution of 1/100. Secondary antibodies anti-mouse IgG H+L and anti-rabbit IgG H+L (Alexa Fluor 647, Life Technologies, Gaithersburg, MD) were applied at 1/2000 dilution. Cells were washed twice in FC buffer between each incubation step, and after adding secondary Ab, they were resuspended in FC-buffer for FC. Twenty thousand events were collected using the Gallios Flow Cytometer (Beckman Coulter, Miami, FL, USA) and analyzed with FlowJo Software (Tree Star, Ashland, OR, USA). Cells infected with MVA-Venus used as a control were first incubated with the primary Ab, followed by the secondary Ab. GFP expression from MVA recombinants, all containing a GFP expression cassette (Figure 1B and 1C) [46] was analyzed as a control to confirm MVA infection of the investigated cells (data not shown).

### Immunofluorescence

Monolayers of BHK cells (80–90% confluent) were infected with MVA recombinants at MOI of 0.01 and incubated for 16–20 h. Cells were fixed in 2% paraformaldehyde in PBS, and for permeabilization treated with 0.2% Triton-X in PBS. Blocking and staining with primary and secondary Ab was performed for 1 hour in PBS containing 10% FBS. Hybridoma supernatants of the primary Ab anti-HCMV-gH mAb 14-4b from mouse were applied at 1/10 dilution. Rabbit anti-Histone H3 (D1H2) mAb (Cell Signaling, Danvers, MA) was used for IF according to the manufacturer's instructions. Fluorophore-coupled secondary antibodies anti mouse IgG Alexa Fluor 555 and anti-rabbit IgG Alexa Fluor 647 (Life Technologies) were used at 1/500 dilution. Cell nuclei were stained for 2–5 min with DAPI diluted in PBS. Cells were washed 2–3 times with PBS after each incubation step. Immunofluorescence was imaged by the Zeiss LSM510 META NLO Axiovert 200 M Inverted Microscope (Carl Zeiss, Jena, Germany). Images were processed with LSM Image Browser from Zeiss.

### Mouse vaccination

Groups of four or eight BALB/cJ mice (Jackson Laboratory, Bar Harbor, ME, USA) were vaccinated three times with 100 µl of 5×10^7^ PFU of purified MVA by i. p. injection from the abdominal side into the body cavity. This injection method was chosen because of experience with this procedure. Blood samples were collected by eye bleed. All mouse handling procedures were approved by the City of Hope Institutional Animal Care and Use Committee (IACUC).

### Monkey vaccination

Groups of four RM were vaccinated three times six weeks apart by intramuscular injection with ∼5×10^8^ PFU of purified MVA as described [111]. Blood samples and oral swabs to determine NAb titer were prepared as described [111]. Monkeys used in this study were genetically outbred RM (*Macaca mulatta*) from the California National Primate Research Center (CNPRC), repeatedly confirmed to be RhCMV seronegative to prevent cross-reactivity with HCMV in downstream neutralization assays. Their age was ∼1–2 years at the time of vaccination. The animals were co-housed in pairs at least two weeks before vaccination, and remained co-housed until study completion. The IACUC of the University of California, Davis (UC Davis), which is fully accredited by the Association for Assessment and Accreditation of Laboratory Animal Care, approved all animal protocols.

### Neutralization assays

HCMV microneutralization assay was performed similar to published reports [66]. Heat-inactivated sera were serially two-fold diluted in 100 µl volumes using complete growth medium for ARPE-19 cells, MRC-5 fibroblasts, HUVECs, or HC depending on the cell type used in the assay. Dilutions ranged from 1∶25 to 1∶102400. Diluted serum was mixed with 100 µl of complete growth medium containing approximately 2400 PFU of HCMV. After 2 h incubation, mixtures were added in triplicate (50 µl) to cells (ARPE-19 cells, MRC-5 fibroblasts, HUVECs, or HC) seeded the day before at 1.5×10^4^ cells/well in a clear bottom Polystyrene 96-well plate (Corning) that contained 50 µl per well of complete growth medium. Cells were grown for 48 h and fixed in methanol/acetone. Infected cells were identified by immunostaining using mouse anti-HCMV IE1 Ab (p63-27) and the Vectastain ABC kit (VectorLabs, Burlingame, CA, USA). The substrate was 3, 3′-diaminobenzidine (DAB, VectorLabs). Plates were analyzed by an automated system using the Axio Observer Z1 inverted microscope equipped with a linear motorized stage (Carl Zeiss). IE1 positive nuclei were counted using ImagePro Premier (Media Cybernetics, Silver Spring, MD, USA). For each dilution the average number of positive nuclei in triplicate was calculated. The percent neutralization titer (NT) for each dilution was calculated as follows: NT = [1−(positive nuclei number with immune sera)/(positive nuclei number with pre-immune sera)]×100. The titers that gave 50% neutralization (NT50) or 90% neutralization (NT90) were calculated by determining the linear slope of the graph plotting NT versus plasma dilution by using the next higher and lower NT values that were closest to 50% or 90% neutralization respectively.

### Human sera and IgG

HCMV IgG positive and negative sera were derived from a pool of seven plasma bags that were donated by volunteers and tested for HCMV IgG by the City of Hope Blood Bank. Commercially available HCMV seropositive (Lots BM204234, BM204360 and BM204371) and negative (Lot BM216642) sera were purchased from SeraCare Life Sciences (Oceanside, CA, USA). Intravenous human immunoglobulin (IVIg) was purchased from Privigen, CSL Behring (Marburg, Germany). Cytogam (CMV-IVIg) was obtained from the manufacturer (Baxter-Healthcare Corp., Irvine, CA, USA).

### HCMV-TB40/E antigen

HCMV antigen for ELISA assays was prepared as follows. Approximately 2.5×10^7^ ARPE-19 cells showing 80–90% CPE after HCMV TB40/E infection were harvested in 5 ml of glycine-saline buffer (0.04 M Glycine, 0.15 M NaCl, 0.01 M NaOH, pH 9). Cell suspensions were incubated on ice for 4 h. Cells were dounce-homogenized and cell lysates were clarified at 1500×g at 4°C. Supernatants were aliquoted and stored at −80°C until use.

### HCMV IgG ELISA

Microtiter wells (EIA/RIA Plates, Costar, Corning) were coated overnight with 100 µl HCMV TB40/E antigen prepared as described above. Wells were washed three times with PBS and blocked for 2 h with PBS containing 1% BSA. Wells were washed again in PBS containing 0.1% Tween20 (wash-buffer) and serum samples diluted 1/100 in sample buffer (PBS, 0.1% Tween 20, 1% BSA) were added in quadruplicate and incubated for 2 h. Two wells of each quadruplicate were washed either in wash-buffer or wash buffer containing 6 M urea; then, all wells were washed again two times in wash-buffer. Wells were incubated with secondary anti-mouse HRP conjugate (Promega) or anti-monkey HRP conjugate (KPL) and then developed with TMB substrate reagent. OD values at 450 were measured and normalized based on week zero OD. Avidity index (AI) values were expressed as percentage of the ratio of OD in wells washed with wash buffer containing 6 M urea/OD in wells washed with wash buffer.

### Oral swab total and HCMV-specific IgG

Evaluation of total IgG titer in oral swab samples was performed using an ELISA kit for human/monkey IgG (Mabtech, Stockholm, Sweden) according to the manufacturer's instructions. Oral swab samples were diluted 1/240 in sample buffer (PBS, 0.1% Tween 20, 1% BSA). Standard or sample dilutions were added in duplicate. Total IgG concentration was derived from the standard curve. HCMV-specific IgG assay was performed as above using CMV-TB40/E antigen coated plates. Oral swab samples were diluted 1/24 in sample buffer and added in duplicate. CMV-IgG levels were expressed as OD at 405 nm.

### Oral swab total and CMV-specific IgA

Evaluation of total IgA titer in oral swab samples was performed using ELISA for monkey IgA (Mabtech) according to the manufacturer's instructions. For the assay, oral swab samples were diluted 1/240 in sample buffer. Standard or sample dilutions were added in duplicate wells. Total IgA concentration was derived from the standard curve. CMV-specific IgA assay was performed as above but using CMV-TB40/E antigen coated plates. Oral swab samples were diluted 1/24 in sample buffer and added in duplicate wells. CMV-IgA levels were expressed as OD at 405 nm.

## Supporting Information

Figure S1 **Cell surface imaging of gH expressed from MVA recombinants.** Monolayers of BHK cells were infected at a low multiplicity of infection with the indicated MVA recombinants (MVA-gH/gL-PC, MVA-gH/gL-PCΔ, and MVA-gH/gL). Cells were fixed 16 h after the infection and either left untreated (non-permeabilized) or permeabilized. Staining was performed with mouse anti-HCMV gH mAb 14-4b and anti-mouse Alexa Flour 555 secondary Ab. Cell nuclei were stained with DAPI. Immunofluorescence was imaged by confocal microscopy. GFP expression from the MVA vectors (all BAC-reconstituted MVA expressed GFP due to the vector construction (Figure 1) was detected to localize foci of virus spread. Staining of Histone H3 using anti-Histone H3 rabbit mAb and secondary Alexa Flour 647 Ab was performed as a control to determine if non-permeabilized cells were non-penetrable by antibodies and permeabilized cells were penetrable by antibodies.
(TIF)

Figure S2 **Distance tree of different HCMV clinical and laboratory strains.** Protein sequences of different HCMV laboratory strains (AD169, Davis and Towne) and clinical isolates that have been passaged to a limited extent in the laboratory (Merlin, TB40/E, Toledo, TR and VR1814) were analyzed using COBALT multiple alignment tool and a phylogenetic tree was build using Fast Minimum Evolution algorithm and applying a 0.85 maximum sequence difference and the Grishin evolutionary distance model.
(TIF)

Figure S3 **Salivary IgG and IgA responses in individual RM vaccinated with MVA-gH/gL-PC or MVA-gH/gL.** Oral swab samples from immunized RM were analyzed using commercial IgG/IgA ELISA kits for measuring total Ab levels. IgG/IgA ELISA assays using CMV TB40E antigen coated plates were used to determine CMV-specific IgG/IgA levels. **A and B)** Oral swab mucosal IgA levels in RM vaccinated with MVA-gH/gL-PC or MVA-gH/gL. **C and D)** CMV-mucosal IgA levels in oral swabs from MVA-gH/gL-PC or MVA-gH/gL vaccinated RM. **E and F)** Oral swab IgG levels in RM vaccinated with MVA-gH/gL-PC or MVA-gH/gL. **G and H)** CMV-IgG levels in oral swabs from MVA-gH/gL-PC or MVA-gH/gL vaccinated RM. Dotted lines in A, B, E and F represent the detection limit of the assay, dashed lines in C, D, G and H represent the upper limit of the 99% confidence interval for the mean values obtained in the MVA-Venus RM. Filled triangles indicate MVA injections.
(TIF)

Figure S4 **Binding antibodies (BAb) of HCMV proteins in sera from vaccinated animals.** Serum preparations from mice and monkeys vaccinated with MVA recombinants (MVA-gH/gL-PC, MVA-gH/gL-PCΔ, MVA-gH/gL, MVA-UL128-131, MVA-gB, MVA-gBΔ, MVA-Venus) were used at a dilution of 1/15000 to detect gH, gL, UL128, UL130, UL131A or gB expressed from Ad vectors in ARPE-19 cells by WB (see Material and Methods for detailed WB description). Ad tet-trans was analyzed as a control. Shown are WBs using one representative serum sample from one mouse or RM per vaccine group (see Figure 3 and 4 for vaccine groups) obtained after 3 MVA vaccinations (mice) or 2 MVA immunizations (RM) (see Figure 3 and 4 for vaccination timelines). Arrows indicate the expected protein bands. **A)** BAb in MVA vaccinated BALB/cJ mice. **B)** BAb in RM vaccinated with MVA recombinants.
(TIF)

Table S1 **Analysis of mouse serum NT50 levels on ARPE-19, MRC-5 fibroblasts and HUVECs after 3 MVA vaccinations.** Groups of 4 BALB/c mice were vaccinated 3 times at week 0, 4 and 8 using the MVA constructs shown in the table. NAb levels were evaluated on ARPE-19, MRC-5 fibroblasts and HUVECs using serum samples collected at 3, 7, 11 and 16 weeks after the first vaccination. Shown in the table is the average serum NT50 and standard deviation.
(DOCX)

Table S2 **Analysis of RM serum NT50 levels on ARPE-19, MRC-5 fibroblasts and HUVECs after 3 MVA vaccinations.** Groups of 4 RM were vaccinated 3 times at week 0, 6 and 12 with MVA-gH/gL-PC, MVA-gH/gL or MVA-Venus. NAb levels were evaluated on ARPE-19, MRC-5 fibroblasts and HUVECs using serum samples collected at different time points (Figure 6A). Listed in the table are individual animal measurements and group average NT50.
(DOCX)

Table S3 **RM Serum NT90 levels measured on ARPE-19 cells and HC after 2 MVA vaccinations.** Shown in the table is the serum NT90 obtained on ARPE-19 and HC using RM serum collected 8 weeks after the first vaccination.
(DOCX)

Table S4 **Analysis of saliva NT50 levels in MVA-gH/gL-PC vaccinated RM measured on ARPE-19 cells.** The table shows longitudinal variation of NT50 titers in saliva samples of individual RM measured on ARPE-19 cells against HCMV TB40/E.
(DOCX)

Table S5 **Primer list.** Shown in the table is the list of primers used to generate the transfer vectors for gene insertion into the Del2, IGR3, G1L and I8R of the MVA-BAC (See Materials and Methods for details).
(DOCX)
