# Supplementary figures and images for: Human Cytomegalovirus Vaccine Based on the Envelope gH/gL Pentamer Complex
Source: PLoS Pathog. 2014 Nov 20;10(11):e1004524. doi: 10.1371/journal.ppat.1004524 (PMC4239111; doi:10.1371/journal.ppat.1004524)

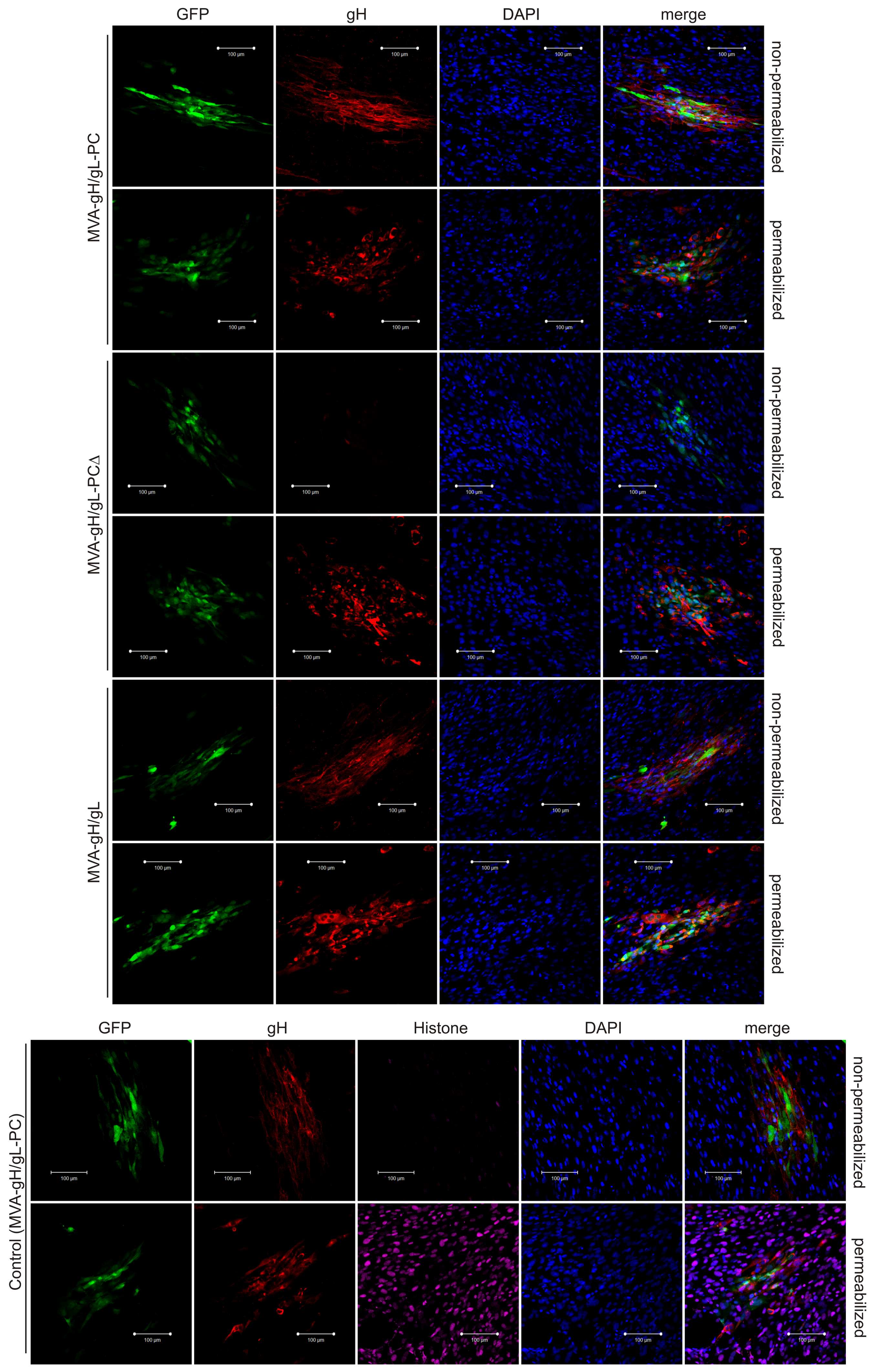

Supplement: Figure S1 — Cell surface imaging of gH expressed from MVA recombinants. Monolayers of BHK cells were infected at a low multiplicity of infection with the indicated MVA recombinants (MVA-gH/gL-PC, MVA-gH/gL-PCΔ, and MVA-gH/gL). Cells were fixed 16 h after the infection and either left untreated (non-permeabilized) or permeabilized. Staining was performed with mouse anti-HCMV gH mAb 14-4b and anti-mouse Alexa Flour 555 secondary Ab. Cell nuclei were stained with DAPI. Immunofluorescence was imaged by confocal microscopy. GFP expression from the MVA vectors (all BAC-reconstituted MVA expressed GFP due to the vector construction (Figure 1) was detected to localize foci of virus spread. Staining of Histone H3 using anti-Histone H3 rabbit mAb and secondary Alexa Flour 647 Ab was performed as a control to determine if non-permeabilized cells were non-penetrable by antibodies and permeabilized cells were penetrable by antibodies. (TIF) [file ppat.1004524.s001.tif]

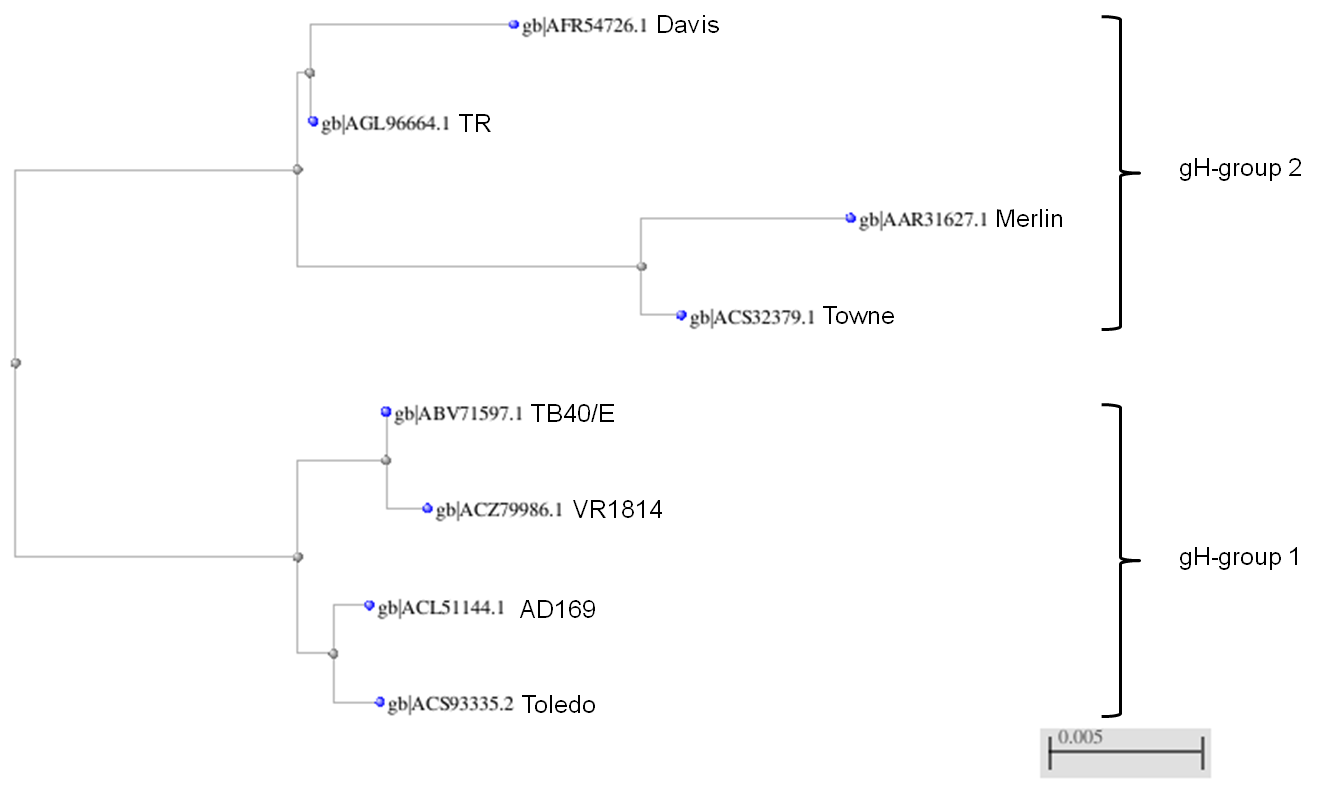

Supplement: Figure S2 — Distance tree of different HCMV clinical and laboratory strains. Protein sequences of different HCMV laboratory strains (AD169, Davis and Towne) and clinical isolates that have been passaged to a limited extent in the laboratory (Merlin, TB40/E, Toledo, TR and VR1814) were analyzed using COBALT multiple alignment tool and a phylogenetic tree was build using Fast Minimum Evolution algorithm and applying a 0.85 maximum sequence difference and the Grishin evolutionary distance model. (TIF) [file ppat.1004524.s002.tif]

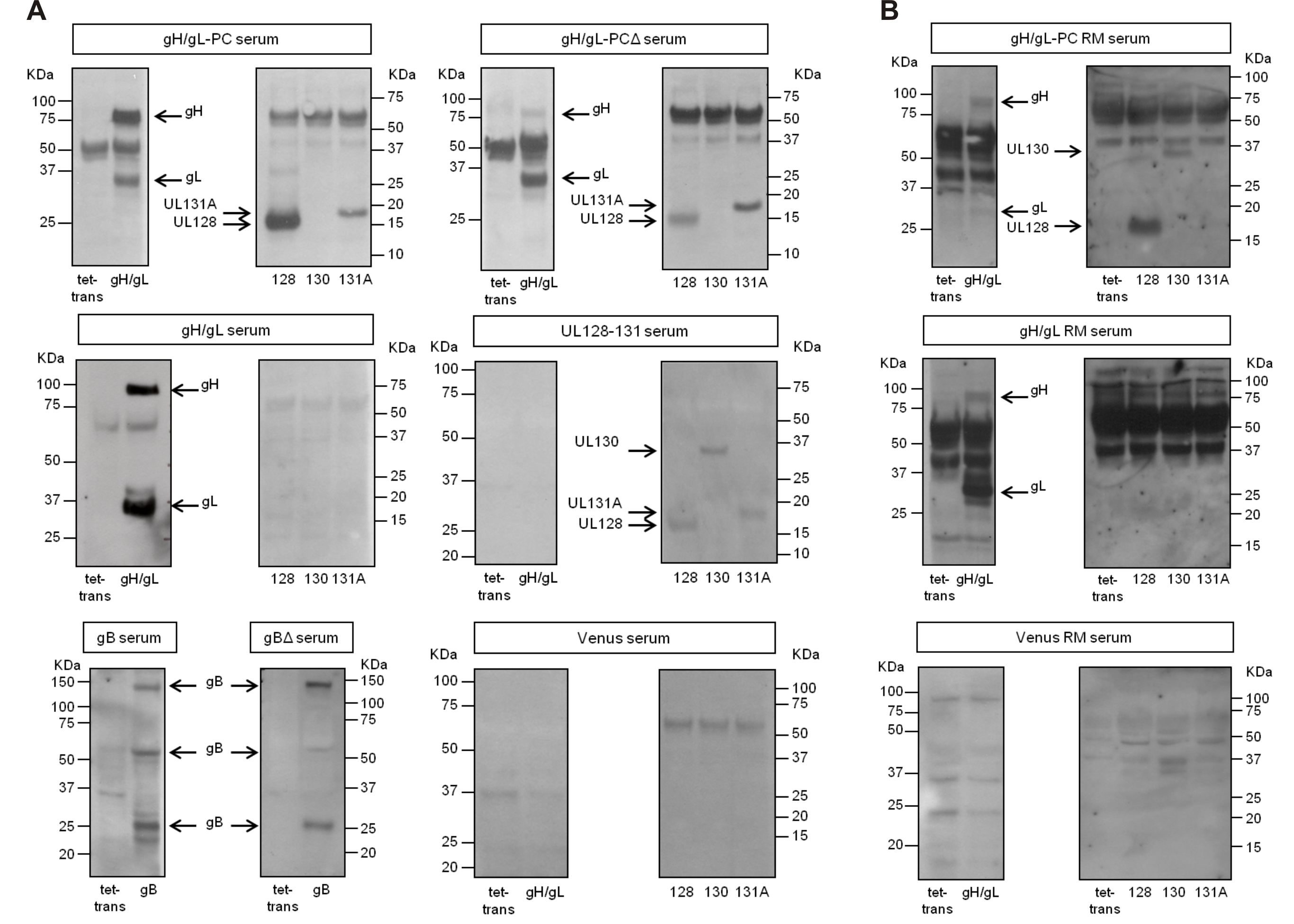

Supplement: Figure S4 — Binding antibodies (BAb) of HCMV proteins in sera from vaccinated animals. Serum preparations from mice and monkeys vaccinated with MVA recombinants (MVA-gH/gL-PC, MVA-gH/gL-PCΔ, MVA-gH/gL, MVA-UL128-131, MVA-gB, MVA-gBΔ, MVA-Venus) were used at a dilution of 1/15000 to detect gH, gL, UL128, UL130, UL131A or gB expressed from Ad vectors in ARPE-19 cells by WB (see Material and Methods for detailed WB description). Ad tet-trans was analyzed as a control. Shown are WBs using one representative serum sample from one mouse or RM per vaccine group (see Figure 3 and 4 for vaccine groups) obtained after 3 MVA vaccinations (mice) or 2 MVA immunizations (RM) (see Figure 3 and 4 for vaccination timelines). Arrows indicate the expected protein bands. A) BAb in MVA vaccinated BALB/cJ mice. B) BAb in RM vaccinated with MVA recombinants. (TIF) [file ppat.1004524.s004.tif]
