# Supplementary material for: Human Cytomegalovirus Vaccine Based on the Envelope gH/gL Pentamer Complex
Source: PLoS Pathog. 2014 Nov 20;10(11):e1004524. doi: 10.1371/journal.ppat.1004524 (PMC4239111; doi:10.1371/journal.ppat.1004524)
Supplement: Table S1 — Analysis of mouse serum NT50 levels on ARPE-19, MRC-5 fibroblasts and HUVECs after 3 MVA vaccinations. Groups of 4 BALB/c mice were vaccinated 3 times at week 0, 4 and 8 using the MVA constructs shown in the table. NAb levels were evaluated on ARPE-19, MRC-5 fibroblasts and HUVECs using serum samples collected at 3, 7, 11 and 16 weeks after the first vaccination. Shown in the table is the average serum NT50 and standard deviation. (DOCX) [file ppat.1004524.s005.docx]

|  |  |  |  |  |  |  |  |  |  |  |  |  |  |
| --- | --- | --- | --- | --- | --- | --- | --- | --- | --- | --- | --- | --- | --- |
| **Table S1.** **BALB/C mouse NT50 levels on ARPE-19, MRC-5 and HUVECs after 3 MVA vaccinations** | | | | | | | | | | | | | |
|  |  |  | **1st^A^** | |  | **2nd^A^** | |  | **3rd^A^** | | | | |
| **Cell Type** | **MVA** |  | **3Wk**^B^ | |  | **7Wk**^B^ | |  | **11Wk**^B^ | |  | **16Wk**^B^ | |
|  |  |  | **Ø^C^** | **SD^D^** |  | **Ø^C^** | **SD^D^** |  | **Ø^C^** | **SD^D^** |  | **Ø^C^** | **SD^D^** |
|  | **gH/gL-PC** |  | 5970 | 2710 |  | 62520 | 18270 |  | 31280 | 11640 |  | 30010 | 11410 |
|  | **gH/gL-PCΔ** |  | <100 | 0 |  | 5960 | 3490 |  | 62540 | 18720 |  | 47000 | 26110 |
| **ARPE-19** | **gH/gL** |  | 370 | 90 |  | 2170 | 2510 |  | 1290 | 1740 |  | 560^┼^ | 370 |
|  | **UL128-131** |  | <100 | 0 |  | <100 | 0 |  | <100 | 0 |  | <100 | 0 |
|  | **gB** |  | <100 | 0 |  | <100 | 0 |  | <100 | 0 |  | <100 | 0 |
|  | **gBΔ** |  | <100 | 0 |  | 240 | 170 |  | 250 | 170 |  | <100 | 0 |
|  | **gH/gL-PC** |  | 600 | 390 |  | 1180 | 670 |  | 760 | 640 |  | 600 | 580 |
|  | **gH/gL-PCΔ** |  | <100 | 0 |  | 310 | 140 |  | 740 | 460 |  | 500 | 340 |
| **MRC-5** | **gH/gL** |  | 430 | 120 |  | 1120 | 1080 |  | 680 | 540 |  | 330^┼^ | 160 |
|  | **UL128-131** |  | <100 | 0 |  | <100 | 0 |  | <100 | 0 |  | <100 | 0 |
|  | **gB** |  | 160 | 90 |  | 530 | 200 |  | 690 | 500 |  | <100 | 0 |
|  | **gBΔ** |  | 190 | 110 |  | 510 | 540 |  | 250 | 190 |  | <100 | 0 |
|  | **gH/gL-PC** |  | -^E^ | -^E^ |  | 58280 | 20800 |  | -^E^ | -^E^ |  | 26200 | 7810 |
|  | **gH/gL-PCΔ** |  | -^E^ | -^E^ |  | 5120 | 2350 |  | -^E^ | -^E^ |  | 46260 | 20210 |
| **HUVEC** | **gH/gL** |  | -^E^ | -^E^ |  | 2700 | 3340 |  | -^E^ | -^E^ |  | 527^┼^ | 230 |
|  | **UL128-131** |  | -^E^ | -^E^ |  | <100 | 0 |  | -^E^ | -^E^ |  | <100 | 0 |
|  | **gB** |  | -^E^ | -^E^ |  | <100 | 0 |  | -^E^ | -^E^ |  | <100 | 0 |
|  | **gBΔ** |  | -^E^ | -^E^ |  | 155 | 64 |  | -^E^ | -^E^ |  | <100 | 0 |
| ^A^Vaccination number, ^B^Weeks after first vaccination, ^C^Average NAb titer, ^D^Standard deviation, , ^E^Not available ^┼^One dead mouse | | | | | | | | | | | | | |
