# Supplementary material for: Human Cytomegalovirus Vaccine Based on the Envelope gH/gL Pentamer Complex
Source: PLoS Pathog. 2014 Nov 20;10(11):e1004524. doi: 10.1371/journal.ppat.1004524 (PMC4239111; doi:10.1371/journal.ppat.1004524)
Supplement: Table S2 — Analysis of RM serum NT50 levels on ARPE-19, MRC-5 fibroblasts and HUVECs after 3 MVA vaccinations. Groups of 4 RM were vaccinated 3 times at week 0, 6 and 12 with MVA-gH/gL-PC, MVA-gH/gL or MVA-Venus. NAb levels were evaluated on ARPE-19, MRC-5 fibroblasts and HUVECs using serum samples collected at different time points (Figure 6A). Listed in the table are individual animal measurements and group average NT50. (DOCX) [file ppat.1004524.s006.docx]

| **Table S2. RM Serum NT50 levels measured on ARPE-19, MRC-5 and HUVECs after 3 MVA vaccinations** | | | | | | | | | | | | | | |
| --- | --- | --- | --- | --- | --- | --- | --- | --- | --- | --- | --- | --- | --- | --- |
| **Cell Type** | **MVA** | **RM** |  | **1st^A^** |  | **2nd^A^** | | |  | **3rd^A^** | | | | |
|  |  |  |  | **6Wk^B^** |  | **7Wk^B^** | **8Wk^B^** | **12Wk^B^** |  | **13Wk^B^** | **14Wk^B^** | **18Wk^B^** | **22Wk^B^** | **26Wk^B^** |
|  |  | **RM1** |  | 1860 |  | 78340 | 75280 | 20070 |  | 62730 | 45120 | 9650 | 2960 | 2120 |
|  |  | **RM2** |  | 370 |  | 54490 | 40450 | 4710 |  | 19730 | 12950 | 1630 | 940 | 910 |
|  | **gH/gL-PC** | **RM3** |  | 620 |  | 29960 | 8770 | 1860 |  | 20620 | 10430 | 1490 | 1060 | 880 |
|  |  | **RM4** |  | 1020 |  | -^D^ | 24680 | 10700 |  | 33610 | 20990 | 5000 | 2070 | 1080 |
| **ARPE-19** |  | **Ø^C^** |  | 968 |  | 54263 | 37295 | 9335 |  | 34173 | 22373 | 4443 | 1758 | 1248 |
|  |  | **RM5** |  | <50 |  | 150 | 120 | <50 |  | <50 | 110 | <50 | <50 | <50 |
|  |  | **RM6** |  | <50 |  | 150 | 80 | <50 |  | 60 | <50 | <50 | <50 | <50 |
|  | **gH/gL** | **RM7** |  | <50 |  | 180 | 350 | <50 |  | 90 | 60 | <50 | <50 | <50 |
|  |  | **RM8** |  | <50 |  | 530 | 250 | <50 |  | 350 | 180 | <50 | <50 | <50 |
|  |  | **Ø**^C^ |  | <50 |  | 253 | 200 | <50 |  | 138 | 100 | <50 | <50 | <50 |
|  |  | **RM1** |  | <25 |  | 630 | 470 | 160 |  | 180 | 100 | <25 | <25 | <25 |
|  |  | **RM2** |  | <25 |  | 300 | 60 | <25 |  | 60 | 50 | <25 | <25 | <25 |
|  | **gH/gL-PC** | **RM3** |  | <25 |  | 190 | 140 | <25 |  | 90 | 70 | <25 | <25 | <25 |
|  |  | **RM4** |  | <25 |  | -^D^ | 250 | 140 |  | 150 | 160 | 30 | <25 | <25 |
| **MRC-5** |  | **Ø^C^** |  | <25 |  | 373 | 230 | 88 |  | 120 | 95 | 25 | <25 | <25 |
|  |  | **RM5** |  | <25 |  | 310 | 270 | 30 |  | 150 | 170 | <25 | <25 | <25 |
|  |  | **RM6** |  | <25 |  | 100 | 60 | <25 |  | 60 | 40 | <25 | <25 | <25 |
|  | **gH/gL** | **RM7** |  | <25 |  | 170 | 150 | <25 |  | 40 | <25 | <25 | <25 | <25 |
|  |  | **RM8** |  | <25 |  | 310 | 290 | <25 |  | <25 | <25 | <25 | <25 | <25 |
|  |  | **Ø**^C^ |  | <25 |  | 223 | 193 | 25 |  | 69 | 65 | <25 | <25 | <25 |
|  |  | **RM1** |  | -^E^ |  | -^E^ | 80000 | -^E^ |  | -^E^ | -^E^ | -^E^ | -^E^ | 1960 |
|  |  | **RM2** |  | -^E^ |  | -^E^ | 47230 | -^E^ |  | -^E^ | -^E^ | -^E^ | -^E^ | 430 |
|  | **gH/gL-PC** | **RM3** |  | -^E^ |  | -^E^ | 8220 | -^E^ |  | -^E^ | -^E^ | -^E^ | -^E^ | 1240 |
|  |  | **RM4** |  | -^E^ |  | -^E^ | 18580 | -^E^ |  | -^E^ | -^E^ | -^E^ | -^E^ | 2400 |
| **HUVEC** |  | **Ø^C^** |  | -^E^ |  | -^E^ | 38510 | -^E^ |  | -^E^ | -^E^ | -^E^ | -^E^ | 1510 |
|  |  | **RM5** |  | -^E^ |  | -^E^ | 400 | -^E^ |  | -^E^ | -^E^ | -^E^ | -^E^ | <25 |
|  |  | **RM6** |  | -^E^ |  | -^E^ | 70 | -^E^ |  | -^E^ | -^E^ | -^E^ | -^E^ | <25 |
|  | **gH/gL** | **RM7** |  | -^E^ |  | -^E^ | 170 | -^E^ |  | -^E^ | -^E^ | -^E^ | -^E^ | <25 |
|  |  | **RM8** |  | -^E^ |  | -^E^ | 360 | -^E^ |  | -^E^ | -^E^ | -^E^ | -^E^ | <25 |
|  |  | **Ø**^C^ |  | -^E^ |  | -^E^ | 250 | -^E^ |  | -^E^ | -^E^ | -^E^ | -^E^ | <25 |
| ^A^Number of vaccinations, ^B^Weeks after initial vaccination, ^C^Average NT50, ^D^Missing sample, ^E^Not available | | | | | | | | | | | | | | |
