# Supplementary material for: Human Cytomegalovirus Vaccine Based on the Envelope gH/gL Pentamer Complex
Source: PLoS Pathog. 2014 Nov 20;10(11):e1004524. doi: 10.1371/journal.ppat.1004524 (PMC4239111; doi:10.1371/journal.ppat.1004524)
Supplement: Table S3 — RM Serum NT90 levels measured on ARPE-19 cells and HC after 2 MVA vaccinations. Shown in the table is the serum NT90 obtained on ARPE-19 and HC using RM serum collected 8 weeks after the first vaccination. (DOCX) [file ppat.1004524.s007.docx]

**Table S3.** **RM Serum NT90 levels measured on ARPE-19 cells and HC after 2 MVA vaccinations**

| **Cell type** |  | **MVA-gH/gL-PC** | | | |  | **MVA-gH/gL** | | | |  | **MVA-Venus** | | | |
| --- | --- | --- | --- | --- | --- | --- | --- | --- | --- | --- | --- | --- | --- | --- | --- |
|  |  | **RM1** | **RM2** | **RM3** | **RM4** |  | **RM5** | **RM6** | **RM7** | **RM8** |  | **RM9** | **RM10** | **RM11** | **RM12** |
| **ARPE-19** |  | 37500 | 16280 | 4030 | 8650 |  | <25 | <25 | <25 | <25 |  | <25 | <25 | <25 | <25 |
| **HC** |  | 7070 | 4460 | 1770 | 3800 |  | <25 | <25 | <25 | <25 |  | <25 | <25 | <25 | <25 |
