# Supplementary material for: Human Cytomegalovirus Vaccine Based on the Envelope gH/gL Pentamer Complex
Source: PLoS Pathog. 2014 Nov 20;10(11):e1004524. doi: 10.1371/journal.ppat.1004524 (PMC4239111; doi:10.1371/journal.ppat.1004524)
Supplement: Table S4 — Analysis of saliva NT50 levels in MVA-gH/gL-PC vaccinated RM measured on ARPE-19 cells. The table shows longitudinal variation of NT50 titers in saliva samples of individual RM measured on ARPE-19 cells against HCMV TB40/E. (DOCX) [file ppat.1004524.s008.docx]

| **Table S4.** **Saliva NT50 levels in MVA-gH/gL-PC vaccinated RM measured on ARPE-19 cells** | | | | | | | | | | | |  |
| --- | --- | --- | --- | --- | --- | --- | --- | --- | --- | --- | --- | --- |
| **RM** |  | **1st^A^** |  | **2nd^A^** | | |  | **3rd^A^** | | | | |
|  |  | **6Wk^B^** |  | **1Wk^B^** | **2Wk^B^** | **6Wk^B^** |  | **1Wk^B^** | **2Wk^B^** | **6Wk^B^** | **10Wk^B^** | **14Wk^B^** |
| **RM1** |  | <24 |  | 43.2 | <24 | 30 |  | 87 | 72 | <24 | <24 | <24 |
| **RM2** |  | <24 |  | <24 | <24 | <24 |  | <24 | <24 | <24 | <24 | <24 |
| **RM3** |  | <24 |  | <24 | 35.4 | <24 |  | <24 | 99 | 34.8 | <24 | <24 |
| **RM4** |  | <24 |  | -**^c^** | <24 | <24 |  | <24 | <24 | <24 | <24 | <24 |
| ^A^Number of vaccinations, ^B^Weeks after the indicated vaccination, ^c^Missing sample | | | | | | | | | | | |  |
