# Supplementary material for: Human Cytomegalovirus Vaccine Based on the Envelope gH/gL Pentamer Complex
Source: PLoS Pathog. 2014 Nov 20;10(11):e1004524. doi: 10.1371/journal.ppat.1004524 (PMC4239111; doi:10.1371/journal.ppat.1004524)
Supplement: Table S5 — Primer list. Shown in the table is the list of primers used to generate the transfer vectors for gene insertion into the Del2, IGR3, G1L and I8R of the MVA-BAC (See Materials and Methods for details). (DOCX) [file ppat.1004524.s009.docx]

| **Table S5. Primer** | | |
| --- | --- | --- |
| **Primer** | **Sequence (5’ to 3’)** | **Amplicon** |
| P1 | GTAGTCGACTTAAATCAATTAGTACACCGCTATG | Left Del2 flank |
| P2 | GCCGTCGACGCATGCCTCGAGCCTAGGTCGTATTCGATGATTATTTTTAAC |  |
| P3 | GTAACTAGTGAATCATCCAGTCCACTGAATAGC | Right Del2 flank |
| P4 | GCCACTAGTTGCGTCGTTTAAACATATGACTAG |  |
| P5 | GTAGTCGACGATTAGTTTCTTGAGTGCGAATG | Left IGR3 flank |
| P6 | GCCGTCGACAAGCTTCTCGAGCCTAGGATAAAAATCAAATGAGATAAAGTGAAAATATATATC |  |
| P7 | GTAACTAGTGAATAAAAATGTTTTTGTTTAACCAC | Right IGR3 flank |
| P8 | GCCACTAGTAACTAGCTGAGAAGGAAGGACC |  |
| P9 | GTAGTCGACTCTACTCCTTATTTGGAATCCAGC | Left G1L/I8R flank |
| P10 | GCCGTCGACAAGCTTCTCGAGCCTAGGATAAAAATTTTTAGTTATTATCTACAGGAACAAATATAG |  |
| P11 | GTAACTAGTATAAAAATCAAACTCTAATGACCACATC | Right G1L/I8R flank |
| P12 | GCCACTAGTACGCAACCAATGATGGACTG |  |
| P13 | GTCGACGAATCATCCAGTCCACTGAATAGCAAAATCTTTACTATTTTGGTATCTTCTAGGGATAACAGGGTAATCGATTT | *aphAI*-I-SceI for Del2 |
| P14 | GTCGACGCCAGTGTTACAACCAATTAACC |  |
| P15 | GTCGACGAATAAAAATGTTTTTGTTTAACCACTGCATGATGTACAGATTTCGGAATTAGGGATAACAGGGTAATCGATTT | *aphAI*-I-SceI for IGR3 |
| P16 | GTCGACGCCAGTGTTACAACCAATTAACC |  |
| P17 | GTCGACATAAAAATCAAACTCTAATGACCACATCTTTTTTTAGAGATGAAAAATTTTAGGGATAACAGGGTAATCGATTT | *aphAI*-I-SceI for G1L/I8R |
| P18 | GTCGACGCCAGTGTTACAACCAATTAACC |  |
| P19 | GTAACTAGTAAAAATTGAAAATAAATACAAAGGTTC | mH5 promoter |
| P20 | GCCACTAGTATAAAAAGGCGCGCCTGCAGGTACCTAGGATATCTTATTTATGATTATTTCTCGCTTTCA |  |
| P21 | GCGTTTAAACGCCGCCACCATGCTACGGCTTCTGCTTCG | ^#^TB40/E UL130 |
| P22 | AGGCGCGCCTCAAACGATGAGATTGGGATGG |  |
| P23 | GCGTTTAAACGCCGCCACCATGTGCCGCCGCCCGGAT | ^#^TB40/E gL |
| P24 | AGGCGCGCCTTAGCGAGCATCCACTGCTTGAGGG |  |
| P25 | CGGTACCTGAGCGGTCGCAACCAGACCATCCTCCAACGGATGCCCCGAACGTAGGGATAACAGGGTAATCGATTT | *aphAI*-I-SceI for UL130 |
| P26 | CGGTACCGCCAGTGTTACAACCAATTAACC |  |
| P27 | GTACAATTGCGGGCCCTGCTGACACTGTTGAGCTCGGACACAGCGCCGCGCTGTAGGGATAACAGGGTAATCGATTT | *aphAI*-I-SceI for gL |
| P28 | GCCCAATTGGCCAGTGTTACAACCAATTAACC |  |
| P29 | GCGTTTAAACGCCGCCACCATGCGGCCCGGCCTCCCC | ^#^TB40/E gH |
| P30 | AGGCGCGCCTCAGCATGTCTTGAGCATGCGGTAGAGC |  |
| P31 | GCGTTTAAACGCCGCCACCATGCGGCCCGGCCTCCCC | ^#^TB40 gHΔTM with myc-tag |
| P32 | AGGCGCGCCTCAGTTCAGGTCCTCTTCAGAGATCAGCTTCTGCTCACGGCTGTCGGTGGCGTCCAC |  |
| P33 | GCGTTTAAACGCCGCCACCATGGAATCCAGGATCTGGTGC | ^#^TB40/E gB |
| P34 | AGGCGCGCCTCAGACGTTCTCTTCTTCGTCAG |  |
| P35 | GCGTTTAAACGCCGCCACCATGGAATCCAGGATCTGGTGC | ^#^TB40/E gBΔ |
| P36 | AGGCGCGCCTCATTCGAGGTCAAAAACGTTG |  |
| P37 | TTGGGGAAATATGAACCTGACATGATTAAGATTGCTCTTTCGGTGGCTGGCTAGTATAAAAAGGCGCGCC | UL130 for EP mutagenesis |
| P38 | TACGCCAAGCTATTTAGGTGACACTATAGAATACTCAAGCTTGGCCGGCCGAAAAATTGAAAATAAATACAAAGG |  |
| P39 | ACAAAATTATGTATTTTGTTCTATCAACTACCTATAAAACTTTCCAAATACTAGTATAAAAAGGCGCGCC | gL for EP mutagenesis |
| P40 | CAAAGTGGATGAATTCCCAGATCCGGCCTTGCCGGCCTCGAGGGCCGGCCGAAAAATTGAAAATAAATACAAAGG |  |
| *underlined sequences correspond to 50 bp duplication as shown in Figure 1. Sequences are derived from ^#^TB40/E-BAC4 Accession # EF999921. | | |
